# A context-dependent and disordered ubiquitin-binding motif

**DOI:** 10.1007/s00018-022-04486-w

**Published:** 2022-08-16

**Authors:** Jesper E. Dreier, Andreas Prestel, João M. Martins, Sebastian S. Brøndum, Olaf Nielsen, Anna E. Garbers, Hiroaki Suga, Wouter Boomsma, Joseph M. Rogers, Rasmus Hartmann-Petersen, Birthe B. Kragelund

**Affiliations:** 1 Structural Biology and NMR Laboratory, University of Copenhagen, Ole Maaloes Vej 5, 2200 Copenhagen N, Denmark; 2 REPIN, University of Copenhagen, Ole Maaloes Vej 5, 2200 Copenhagen N, Denmark; 3 Department of Computer Science, University of Copenhagen, Universitetsparken 1, 2100 Copenhagen Ø, Denmark; 4 Functional Genomics, University of Copenhagen, Ole Maaloes Vej 5, 2200 Copenhagen N, Denmark; 5 Department of Chemistry, Graduate School of Science, The University of Tokyo, Tokyo, 113-0033 Japan; 6 Department of Drug Design and Pharmacology, University of Copenhagen, Jagtvej 160, 2100 Copenhagen Ø, Denmark; 7 The Linderstrøm Lang Centre for Protein Science, Department of Biology, University of Copenhagen, Ole Maaloes Vej 5, 2200 Copenhagen N, Denmark

**Keywords:** IDP, Ubiquitin, SLiM, Context, UBM, NMR, Cyclic peptide, Deep mutational scanning

## Abstract

Ubiquitin is a small, globular protein that is conjugated to other proteins as a posttranslational event. A palette of small, folded domains recognizes and binds ubiquitin to translate and effectuate this posttranslational signal. Recent computational studies have suggested that protein regions can recognize ubiquitin via a process of folding upon binding. Using peptide binding arrays, bioinformatics, and NMR spectroscopy, we have uncovered a disordered ubiquitin-binding motif that likely remains disordered when bound and thus expands the palette of ubiquitin-binding proteins. We term this motif Disordered Ubiquitin-Binding Motif (DisUBM) and find it to be present in many proteins with known or predicted functions in degradation and transcription. We decompose the determinants of the motif showing it to rely on features of aromatic and negatively charged residues, and less so on distinct sequence positions in line with its disordered nature. We show that the affinity of the motif is low and moldable by the surrounding disordered chain, allowing for an enhanced interaction surface with ubiquitin, whereby the affinity increases ~ tenfold. Further affinity optimization using peptide arrays pushed the affinity into the low micromolar range, but compromised context dependence. Finally, we find that DisUBMs can emerge from unbiased screening of randomized peptide libraries, featuring in de novo cyclic peptides selected to bind ubiquitin chains. We suggest that naturally occurring DisUBMs can recognize ubiquitin as a posttranslational signal to act as affinity enhancers in IDPs that bind to folded and ubiquitylated binding partners.

**Supplementary Information:**

The online version contains supplementary material available at 10.1007/s00018-022-04486-w.

## Introduction

Ubiquitin is a small, globular protein involved in a plethora of cellular pathways where it acts as a posttranslational modifier providing signals that are interpreted in complex ways. By the coordinated action of E1, E2 and E3 enzymes, ubiquitin is covalently attached to lysine residues of the target proteins via its C-terminal carboxyl group. As ubiquitin itself has multiple lysines on its surface, additional ubiquitin molecules can be attached to form chains of ubiquitin on the target protein. Depending on which ubiquitin lysine residues that are modified, different types of both linear and branched ubiquitin chains are formed [1, 2]. The most well-known role of ubiquitylation is in the ubiquitin–proteasome system (UPS), where proteins conjugated to K11 or K48 linked ubiquitin chains are degraded by the 26S proteasome [3]. However, other pathways utilize ubiquitylation of other linkage types, where, as an example, K63-linked ubiquitin chains have been shown to play roles in DNA repair pathways and NF-κB signaling [4–6]. Finally, modification with a single ubiquitin molecule may affect binding partner selection, as shown for the DNA sliding clamp proliferating cell nuclear antigen (PCNA) [7] and the actin-bundling protein, fascin [8], where ubiquitylation can turn interactions *on* and *off*, respectively.

Signaling by ubiquitin is dependent on the recognition of ubiquitin and ubiquitin chains. As the length of the ubiquitin chain and the linkage types determine the signaling output, different ubiquitin-binding domains (UBDs) or motifs (UBMs) can recognize and differentiate between different chain types and lengths [9]. Thus, multiple different types of UBDs exist [10–13]. Most are small, α-helical folded domains, and frequently binding is mediated through a single α-helix embedded in a larger fold, as is the case for the ubiquitin interacting motifs (UIMs), inverted UIMs and ubiquitin associated domains. Nonetheless, some UBDs of other structural classes exist, including certain proteasome subunits [3, 14–17] and the ubiquitin-binding zinc finger (UBZ) domain [18].

Many proteins have well-defined globular structures, like ubiquitin. However, the last ~ 20 years have seen the discovery and characterization of intrinsically disordered proteins (IDPs) and protein regions (here collectively termed IDPs), which lack stable three-dimensional folds. These dynamic proteins have intriguing features as they exist in ensembles of states that are biologically functional, despite their lack of fixed structure [19, 20]. IDPs often function through regulatable interactions with other proteins, RNA or DNA, whereupon some IDPs fold to form a folded and more conventional interaction surface [21], while others in the complex remain fully disordered [22]. IDP interactions often occur through short linear motifs (SLiMs) that are short sequence stretches of conserved residues within an otherwise non-conserved region that alone can bind to one or more specific binding partner(s) [23, 24]. SLiMs are themselves sufficient to interact with a binding partner, but often the context of the remainder of the IDP plays determining roles, fine-tuning the interaction in terms of affinity and specificity [25–27] and providing additional binding sites and potential for allosteric regulation and selection [28]. SLiMs can form secondary structures when bound [29, 30] or remain flexible with little structural adaptation in the bound complex [31], and SLiMs can overlap with other functional modules in an IDP [32–34]. Compared to folded domains, SLiMs are smaller in sequence and display more diversity in the modes of interaction with their binding partners and constitute a compact way of carrying expanded binding competence.

Recent computational work addressing the properties of a subgroup of ubiquitin-binding domains has shown that a set of these can be disordered to some degree in their free state and fold into an α-helix in complex with ubiquitin, analogous with known UBD structures [35]. Additionally, in recent years, unconventional ubiquitin-binding regions in two unrelated IDPs: the deleted in split hand/split foot 1 (Dss1), a proteasome subunit [36, 37] and the ubiquitin-conjugating enzyme E2 Cdc34 [38], have been reported. Both proteins lack known UBDs/UBMs and have no resemblance to other motifs. Two ubiquitin-binding sites (UBS), UBS1 and UBS2 were identified in both proteins, with UBS1 having the highest affinity for ubiquitin. The affinities for ubiquitin were in both cases lower than the generally reported ubiquitin binding affinities of UBMs, which typically range from 20 to 300 μM for monoubiquitin [39, 40, 41]. Still, the interactions mapped by NMR are in both cases located in disordered sequence regions and are functionally important in cell assays [36]. Intriguingly, the interaction sites in Dss1 and Cdc34 did not carry a highly homologous discrete sequence motif but harbored instead common features of aromatic residues and a high negative charge. It is possible that these common features constitute a disordered ubiquitin-binding SLiM. However, neither the exact determinants of this motif nor its frequency in proteins have been systematically addressed, and the structure of the bound state, if any, is unknown. Furthermore, the presence of these ubiquitin-binding sites in disordered regions may result in a broader spectrum of structural propensities, including the possibility that disorder in the UBD can be maintained in complex with ubiquitin.

In the current work, we decomposed the determinants of this novel disordered ubiquitin-binding motif, which we term DisUBM using peptide binding arrays, bioinformatics and NMR spectroscopy, as well as analysis of unbiased peptide screens. We find that the motif relies on aromatic and negatively charged residues and is present in a large range of disordered proteins, many with functions in degradation and transcription. We further discover that the affinity of the motif is highly moldable by the surrounding disordered chain context allowing for an enhanced interaction surface with ubiquitin and increasing the affinity ~ tenfold. Further affinity optimization using peptide arrays pushed the affinity into the low μM range, but compromised context dependence. Expression of high-affinity variants did not impact cellular fitness. Finally, we find DisUBMs in de novo cyclic peptides selected for ubiquitin chain binding, showing that DisUBMs can emerge from unbiased screens of random peptide libraries. Through deep mutational scanning of these cyclic peptides, we confirm the importance of the discovered DisUBM sequence features. We suggest that the natural DisUBMs are general affinity enhancers in IDPs that can bind folded partners, which can be post-translationally modified by ubiquitin.

## Materials and methods

### Expression and purification of ubiquitin

Competent BL21(DE3) *E. coli* cells were transformed with a pMCSG7 plasmid coding for *S. pombe* ubiquitin with an N-terminal 6xhistidine-tag, TEV-cleavage site as well as an N-terminal free cysteine. The cells were grown in LB media with 100 mg/mL ampicillin and ubiquitin expression induced at OD_600_ 0.6 by the addition of 1 mM isopropyl *ß*-d-1-thiogalactopyranoside (IPTG). For the expression of ^15^ N-labeled ubiquitin, M9 minimal media with ^15^N labeled NH_4_Cl was used as growth media. After 4 h, the cells were harvested by centrifugation at 5000×*g* for 15 min at 4 °C. The pellet was resuspended in lysis buffer (50 mM Tris, 10 mM imidazole, 150 mM NaCl, pH 8) and lyzed by French press (Constant systems) at 25 kPSI, centrifuged for 40 min at 20,000×*g*, and the supernatant saved for purification. This was mixed with 5 mL nickel Sepharose resin (GE healthcare) preequilibrated with lysis buffer, incubated on a tilting table for 5 min at room temperature (RT) and applied to a gravity-flow column. The column was washed first with 50 mL washing buffer (50 mM tris, 20 mM imidazole, 1 M NaCl, pH 8) with 5 mM *β*-mercaptoethanol (βME) and then with 50 mL lysis buffer with 5 mM βME. Finally, ubiquitin was eluted with 10 mL elution buffer (50 mM tris, 300 mM imidazole, 150 mM NaCl, pH 8) with 5 mM *β*ME. The volume of the eluate was reduced to 2–3 mL by centrifugation with a 3 kDa cut-off Amicon Ultra-15 centrifugal filter (Merck Millipore) at 4000xg. Finally, the concentrated eluate was purified using a HiLoad (16 × 60) Superdex75 Prep Grade size exclusion column (SEC) (GE Healthcare) equilibrated and run with 20 mM NaH_2_PO_4_, 150 mM NaCl, pH 7.5 with 5 mM βME. Fractions containing pure ubiquitin, confirmed by SDS-PAGE, were pooled.

### Expression and purification of Dss1, Dss1 variants, and SUMO

Dss1 N71C (mentioned as “Dss1 WT” here), Dss1 DWD $({}_{40}{\mathrm{WENNW}}_{45} \to {\mathrm{WEDDWW}})$ and Dss1 DMD $({}_{40}{\mathrm{WENNW}}_{45} \to {\mathrm{WEDDWDLWEDDW}})$ variants were all expressed and purified in the same manner except Dss1 WT, which was kept under reducing conditions during the purification by the addition of 5 mM βME. Competent BL21(DE3) *E. coli* cells were transformed with plasmid coding for Dss1 linked N-terminally with 6xhistidine-small ubiquitin-like modifier (SUMO)-tag purchased from Twist. The cells were grown in LB media with 50 mg/L kanamycin. When OD_600_ reached 1.0, expression was induced by the addition of 0.2 mM IPTG. For the expression of ^13^C, ^15^ N-labeled Dss1, M9 minimal media with ^13^C-labeled glucose and ^15^ N-labeled NH_4_Cl were used as growth media. 4 h after induction, the cells were harvested by centrifugation at 5000×*g* for 15 min at 4 °C, the cell pellet resuspended in lysis buffer and lysed by French press (Constant systems) at 25 kPSI. The cell lysate was centrifuged for 40 min at 20000xg and the supernatant applied to a nickel Sepharose resin (GE healthcare) gravity flow column as with ubiquitin. Fractions containing Dss1 were pooled, added 2 mM dithiothreitol (DTT) and 0.1 mg ULP1 protease and dialyzed using a 3.5 MW cut-off (Spectra/Por) against 1 L Tris buffer (25 mM Tris, 150 mM NaCl, pH 8) O/N. To separate Dss1 from the his_6_-SUMO tag, the dialysate was incubated on the nickel Sepharose gravity flow column for 5 min and the flow-through containing Dss1 collected as well as 5 extra mL of lysis buffer added to the column. Next, the resin was washed with 15 mL washing buffer and the flow-through collected. Finally, the His_6_-SUMO tag was eluted from the column using 10 mL elution buffer. The Dss1 sample was centrifuged at 20,000×*g* for 10 min to remove any insoluble contaminants, and the supernatant loaded on a SOURCE™ 15RPC ST 4.6/100 column (Sigma-Aldrich) using a high-performance liquid chromatography (HPLC) Äkta Purifier system (GE healthcare). Before loading, the column was equilibrated with buffer A_Source_ (50 mM NH_4_HCO_3_, degassed). A gradient from 10 to 40% buffer B_Source_ (50 mM NH_4_HCO_3_, 70% (v/v) acetonitrile) was passed over ~ 50 column volumes (1.66 mL) with a flow rate of 2 mL/min. The fractions containing pure Dss1 were pooled and lyophilized. His_6_-SUMO was further purified using SEC as for ubiquitin.

### Expression and purification of Spd1 and variants

Spd1 and Spd1-4G (L33G, V36G, F55G and Y58G) were expressed and purified from ^15^N-labeled (^15^(NH_4_)_2_SO_4_) of ^15^N and ^13^C labeled (^13^C_6_-glucose) M9 medium as previously described [42]. The Spd1-CTD (Spd1_59-124_) was expressed from a pET24b vector coupled to a His_6_-SUMO tag and purified following standard protocols [43]. The sample was purified further by the means of reverse phase chromatography (RPC). A resource-15 RPC 3 mL was equilibrated with the binding buffer (0.1% trifluoroacetic acid (TFA)*)* over 5 column volumes (CV) before the sample was injected. The sample was then eluted in a gradient from 0 to 40% elution buffer (0.08% TFA, 70% acetonitrile) over 3 CV, and a gradient from 40 to 65% elution buffer over 9 CV and then from 65 to 100% elution buffer over 3 CV, flowrate of 1 mL/min. The protein was lyophilized and re-dissolved in NMR buffer. NMR samples were prepared by adding 10% (v/v) D_2_O, 0.02% (w/v) sodium azide and 0.5 mM sodium trimethylsilylpropanesulfonate (DSS) to 309 μL of a protein solution in 10 mM NaH_2_PO_4_ and 100 mM NaCl (pH 7.4).

### Peptides

Peptides were purchased from TAG Copenhagen. They were N-terminally acetylated and C-terminally amidated to mimic the natural context of the sequence and purified by reversed-phase HPLC to a purity of > 95%.

### Purification of Spd1

Spd1 and Spd1 4G were purified as described in [42].

### Mass spectrometry

Aliquots of protein samples were diluted with MilliQ to a final concentration of 1–20 µM. Trifluoroacetic acid (TFA) was added to a final concentration of 0.1% (*v*/*v*) TFA. The samples were analyzed on a matrix-assisted laser desorption/ionization time of flight mass spectrometer (MALDI-TOF MS) using an α-cyano-4-hydroxycinnamic acid matrix and a Bruker Daltonics Autoflex III Smartbeam spectrometer. All MS spectra were recorded in linear acquisition mode and with positive voltage polarity.

### Membrane-bound peptide array

Two identical membrane-bound peptide arrays, each with 50 peptide spots, were purchased from JPT peptide technologies as PepSpots™ (Germany). Here, peptides are covalently attached to a cellulose membrane with the C-terminal amino acid through a β-alanine. Each peptide spot contained approximately 2.44·10^15^ molecules or 4 nmol peptides. For detection of binding, ubiquitin was labeled at the N-terminal free cysteine with Cy5 maleimide fluorophore (GE Healthcare), which has an emission maximum at 666 nm. Ubiquitin or His_6_-SUMO (50 µL–70 µM) was reduced with 10 times molar excess tris(2-carboxyethyl)phosphine (TCEP), and incubated with one aliquot of 10 µL, 3 mM Cy5 in dimethyl sulfoxide (DMSO) at RT for 2 h. Excess Cy5 was neutralized with 10 µL 14.3 mM βME, and the mixture was separated on a gravity flow Sepharose column (from NanoTemper Monolith Protein Labeling Kit Red-NHS (Amine Reactive)). The column was equilibrated with tris buffered saline with Tween 20 (TBS-T) buffer (20 mM Tris, 150 mM NaCl, 1 mM EDTA, 500 µL/L Tween 20, pH 7). The ubiquitin/SUMO-Cy5 mixture (73.5 µL) was loaded on the column, followed by 426.5 µL TBS-T buffer. To evaluate the concentration of ubiquitin and Cy5, the absorbance at 280 nm and 650 nm of each fraction was measured on a ND-1000 NanoDrop spectrophotometer (Thermo Fischer Scientific). Fractions where the absorbance peaked at both wavelengths simultaneously were pooled and used for the peptide array.

All wash and incubation steps were performed at RT on a rocking table. The membranes were first washed with 25 mL TBS-T buffer two times for 5 min. Next, the membranes were blocked with blocking buffer (TBS-T with 5% (*w*/*v*) skim milk powder) for 1 h and washed for 10 min in TBS-T buffer and the background signal at 650 nm acquired using a Chemidoc imager (Bio-Rad). The Cy5-labeled ubiquitin or His_6_-SUMO was diluted to 0.4 µM with blocking buffer and incubated with the membranes for 1 h. After the incubation, the membranes were washed with TBS-T buffer for five times 5 min and the fluorescence signal at 650 nm acquired on the Chemidoc imager. The fluorescent signal was quantified using the image processing software ImageJ. The intensity from each peptide spot was read using the Microarray Profile plugin. The intensities acquired before adding ubiquitin were subtracted from the signals acquired after. Finally, the mean between the corresponding values from the two membranes was calculated and reported as the signal intensity for each peptide.

### Deep mutational scanning of de novo cyclic peptides.

Saturation mutagenesis DNA libraries for cyclic peptides **Ub2i**, **Ub2ii**, **Ub4i**, and **Ub4ix** were prepared using the method previously described [44]. Aliquots of 200 nM biotinylated ^K48^Ub_2_ and ^K48^Ub_4_ were used as the protein targets, as previously described [45]. Deep mutational scanning using canonical and non-canonical amino acids was carried out as described in [44]. Whereas the previous study analyzed a saturation mutagenesis library for one cyclic peptide only, here mutant libraries for four cyclic peptides were combined and analyzed together as a pool. Data shown are the average from duplicate experiments.

### NMR sample preparation

For interaction of the peptides with ^15^N-ubiquitin, the lyophilized peptides were dissolved in MilliQ water and pH adjusted to 7.5. An aliquot with a volume giving 1 mM peptide at 550 µL was lyophilized and dissolved in 550 µL NMR buffer (20 mM NaH_2_PO_4_, 150 mM NaCl, 5 mM TCEP, 125 µM DSS, 5% (*v*/*v*) D_2_O, pH 7.5) with 100 µM ^15^N-ubiquitin, resulting in 1 mM peptide and 100 µM ubiquitin (molar ration 10:1). For the titrations of ubiquitin with each peptide, except the WT Dss1 peptide, 100 µM ubiquitin in NMR buffer was measured as one end point, and the 1 mM peptide and 100 µM ubiquitin as the other. Then, the peptide sample was titrated into the ubiquitin sample with titration points at 0, 50, 150, 300, 400, 500 and 1000 µM peptide. For the WT peptide, a higher concentration and additional titration points were recorded and a 50:1 ration sample (100 µM ubiquitin and 5 mM peptide) used as end point. The 5 mM peptide sample was titrated with the ubiquitin NMR sample to concentrations of 4.44, 4, 3.5, 3, 2.5, 2, 1.5, 1 and 0.5 mM peptide. NMR samples were centrifuged 13.000×*g* for 5 min and transferred to either single-use 5 mm NMR tubes (Bruker) or 3 mm (Shigemi). To prepare NMR samples of ^15^ N-labeled ubiquitin with full-length Dss1 and variants, lyophilized proteins were dissolved directly in the NMR buffer. The variants were each dissolved in 510 µL NMR buffer with 100 µM ubiquitin. The concentration of Dss1 in the NMR sample was determined by measuring the absorbance at 280 nm on a ND-1000 NanoDrop spectrophotometer, calibrated with the NMR buffer with 100 µM ubiquitin. The concentration of Dss1 varied between variants, depending on the purification yield. For all titrations, seven points of varying Dss1 concentration were recorded. To prepare NMR samples of ^15^N-labeled ubiquitin with Spd1, Spd1 was buffer exchanged to MilliQ using a 3 kDa cut-off Amicon Ultra-0.5 ml centrifugal filter (Merk Millipore), lyophilized and resuspended in NMR buffer with 100 µM ubiquitin. NMR samples for carbon detected and ^15^N-R_1_/R_2_ measurement of Dss1 were made from lyophilized ^13^C,^15^N-labeled or ^15^N-labeled Dss1, respectively, dissolved in NMR buffer. For experiments with ubiquitin, 200 µL 1 mM ubiquitin was buffer exchanged to MilliQ water using a 3 kDa cut-off Amicon Ultra-0.5 ml centrifugal filter (Merk Millipore), lyophilized, and subsequently resuspended in 100 µL NMR buffer with 1 mM Dss1 for a final ubiquitin concentration of 2 mM and 1 mM, respectively. All samples were transferred to 3 mm (Shigemi) NMR tubes.

### NMR data acquisition

All NMR spectra were recorded on Bruker Avance III HD 600 MHz spectrometer with a Bruker proton-optimized quadruple resonance NMR ‘inverse’ (QCI) cryoprobe, a Bruker Avance III HD 750 MHz spectrometer with a Bruker proton-optimized triple resonance NMR ‘inverse’ (TCI) cryoprobe and a Bruker Avance Neo 800 MHz spectrometer with a Bruker carbon/nitrogen-optimized triple-resonance NMR 'observe' (TXO) cryoprobe. Assignments of Dss1 in the bound state were done from ^1^H-^15^N-HSQCs, ^1^H-^15^N-HSQC-NOESYs, ^1^H-^15^N-HSQC- TOCSYs, CON, CAN, CBCACON and COCA spectra. Assignment of Spd1 was done using ^1^H-^15^N-HSQC spectra and triple resonance spectra HNCACB CBCACONH, HNCO, HNCACO and HNN. These spectra were recorded on a Varian Unity Inova 750 or 800 ^1^H MHz NMR spectrometers, and the pulse sequences used were from the Varian Biopack. ^1^H chemical shifts were referenced to DSS, and the ^15^N and ^13^C chemical shifts were referenced indirectly using the gyromagnetic ratios. All spectra were zero-filled, apodized using cosine bells in all dimensions, Fourier transformed, and the zero-order phase was corrected manually using either nmrDraw, a component of NmrPipe or qMDD if spectra were recorded using non-linear-sampling (NLS). All spectra were analysed using the Analysis software.

For relaxation measurement recorded at 10 °C*, T*_*2*_ HSQC experiments were repeated eight times with varying delay times: 0.01696 s, 0.03392 s, 0.06784 s, 0.10176 s, 0.13568 s, 0.1696 s, 0.20352 s and 0.27136 s. Three triplicates of the delay series were made in which each repetition had scrambled the sequence of delays to avoid systematic errors. For *T*_*1*_ determinations, HSQC experiments were repeated eight times with varying delay times: 0.02 s, 0.06 s, 0.1 s, 0.2 s, 0.4 s, 0.6 s, 0.8 s and 1.2 s. Three triplicates of the delay series were made in which each repetition had scrambled the sequence of delays to avoid systematic errors. All spectra of Dss1 were recorded at 10 °C. To assign the ^1^H-^15^N-HSQC spectrum of ubiquitin, the spectrum was compared to the BMRB database entry 4769 [46]. As the ubiquitin used in this study had the TEV-cleavage linker, an N-terminal free cysteine as well as the 6xhis tag, ^15^N-NOESY-HSQC (mixing time 120 ms) and ^15^N-TOCSY-HSQC (mixing time 80 ms) spectra were recorded at 25 °C and used for assignments. All spectra were Fourier transformed and processed using NMRPipe [47] except for the CON, CAN, COCA and CBCACON spectra which were processed using the splitcomb command in topspin. The spectra were analyzed using CcpNmr Analysis [48]. Assignments for unbound Dss1 were from [36]. The assignments of backbone atoms of Spd1 have been deposited in the BioMagResBank under the accession number 51376.

To quantify the CSPs of the signals from ubiquitin induced by the addition of Dss1, Dss1 variants, peptides and Spd1, ^1^H,^15^ N-HSQC spectra were recorded and the normalized CSPs from individual peaks calculated using Eq. 1.

1 $$\begin{aligned}&\mathrm{Weighted\, euclidean\, distance}=\mathrm{CSP}=\Delta \delta \\& \quad =\sqrt{{(\Delta \delta H)}^{2}+(\alpha \bullet {\Delta \delta N)}^{2}}. \end{aligned}$$

where *α* is 0.154 [49]. The CSPs were fitted to a one-site-binding model using global fit, and the *K*_*d*_-values are extracted using Eq. 2.

2 $$\Delta \delta \mathrm{obs}=\Delta \delta \mathrm{max}\bullet \frac{\left\{{\left({\left[P\right]}_{t}+{\left[L\right]}_{t}+{K}_{d}\right)-\left[{\left({\left[P\right]}_{t}+{\left[L\right]}_{t}+{K}_{d}\right)}^{2}-4{\left[P\right]}_{t}\bullet {\left[L\right]}_{t}\right]}^\frac{1}{2}\right\}}{2\bullet {\left[P\right]}_{t}}.$$

Peaks giving rise to large uncertainties in the fit, either due to weak signals or overlap, were removed after a first iteration of fitting, and the fit repeated on the remaining dataset.

To calculate the secondary chemical shifts for Dss1, the following equation was used:

3 $$\mathrm{SCS}={\delta }_{\mathrm{obs}}-{\delta }_{\mathrm{random coil}}.$$

where the random coil chemical shift is calculated for the Dss1 sequence using the webtool from [50].

The intensities for each assigned crosspeak in the ^1^H-^15^ N-HSQC spectra from the relaxation experiments were extracted using the seriesTab function in NMRpipe and fitted to the equation:

4 $${I}_{t}={I}_{o}*{e}^{-t*{R}_{1/2}}.$$

Peaks with overlap, severe line broadening or otherwise poor quality were excluded from the analysis. The mean value from triplicates is reported as the *R*_1/2_ values for each Dss1 residue.

### Yeast experiments

The wild-type and *dss1*Δ strains used here have been described before [36]. The Dss1 variants were expressed from the pREP1 plasmid (Genscript). Transformations were performed using LiAc [51]. Growth assays on EMM (Edinburgh minimal media) [52] were performed as described [53]. Co-immunoprecipitations with GFP-trap resin (Chromotek) were performed as previously described [54]. The antibodies were all used at a dilution of 1:1000 and were: anti-Mts4 [55], anti-20S alpha subunits clone MCP231 (Enzo Life Sciences, BML-PW8195), and anti-GFP (Chromotek, 3H9). The secondary antibodies were from Dako Cytomation.

## Results

### Aromatics and negative charges are hot-spots for ubiquitin interaction

**Fig. 1:**
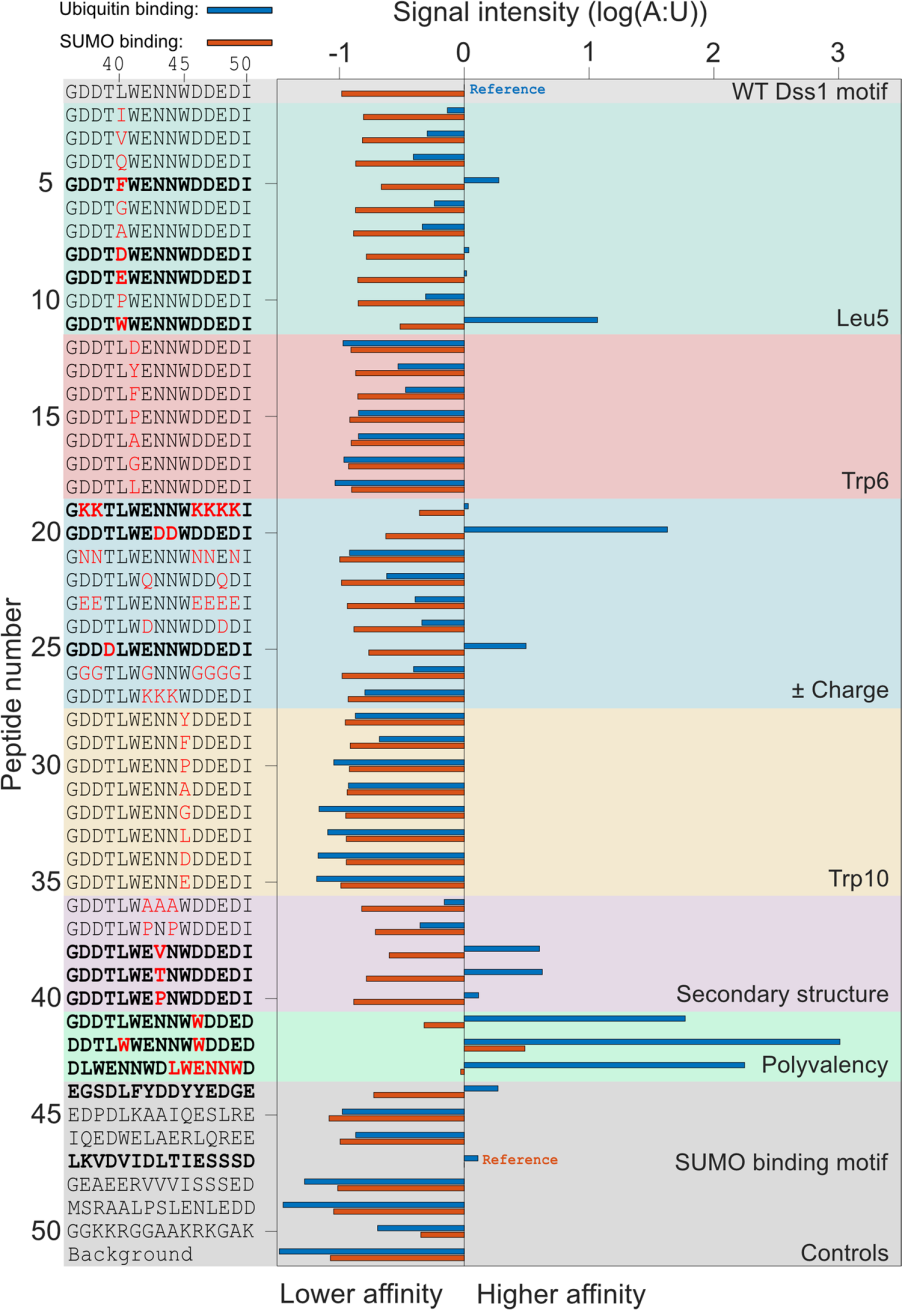
Properties of the disordered ubiquitin-binding motif (DisUBM). Several features of the motif were investigated by a membrane-bound peptide array. The features are indicated to the right (and in different colors), and sequences are shown to the left. Bold sequences indicate peptides with emission intensities larger than the Dss1 UBS1 peptide. The blue and orange bars show emission signals from the peptides incubated with ubiquitin and SUMO, respectively. The emission intensities are normalized to the known binding motifs for ubiquitin (Pep 1) and SUMO (Pep 47), respectively. Ubiquitin binding (blue bars), sumo binding (red bars)

Previous work on Dss1 and Cdc34 has pinpointed ubiquitin binding to occur in disordered regions with little sequence identity, but with common features [36, 38]. Thus, to decompose the determinants for ubiquitin binding, we explored different sequence features of the potential motif through a peptide binding array with 15-mer peptide variants derived from the sequence constituting ubiquitin-binding site 1 (UBS1) in Dss1. A total of 50 different peptides (Pep1-50) were designed to probe various sequence features including the number and kind of charges (Pep19-27), secondary structure propensities (Pep36-40), aromaticity (Pep12-18, Pep28-35) and hydrophobicity (Pep2-11) (Fig. 1). Control peptides (Pep45 and Pep46) with known UIM sequences from vacuolar protein sorting-associated protein 27 (Vps27) and rabaptin-5-associated exchange factor for Rab5 (RABEX5) (*K*_*d*_s of 20 and 100 µM, respectively) [41, 56], as well as the wild-type sequence corresponding to the UBS1 of Dss1 (Pep1; GDDTLW_41_ENNW_45_DDEDI), were included. Recombinant ubiquitin carrying an N-terminal free cysteine was fluorescently labeled and used to probe the peptide array. Excess ubiquitin was removed, and the emission signal corresponding to the bound fraction quantified.

We observed varying fluorescence signals for the different peptides corresponding to different amounts of ubiquitin bound, indicating a diversity in affinities (Fig. 1, blue bars; Fig. S1). The wild-type UBS1 sequence from Dss1 bound ubiquitin as expected, just as the control peptides (Pep45 and Pep46) with sequences from Vps27 and RABEX5, did. However, compared to the wild-type UBS1 peptide from Dss1, the sequences from Vps27 (Pep45) and RABEX5 (Pep46) displayed only weak emission intensities, indicating that their binding affinity for ubiquitin was to some extend compromised in the peptides. Still, the array was able to pick up known ubiquitin binders and allowed us to rank their affinities for ubiquitin based on the fluorescence signals. From an evaluation of emission intensities of the other peptides, several important observations could be made: In the peptide series Pep12-Pep18, Trp41 of the proposed motif was substituted for other types of residues. All substitutions led to a decrease in the detected emission, indicating a lower affinity for ubiquitin, but with phenylalanine and tyrosine substitutions binding stronger than non-aromatic substitutions. A similar result was observed in the Pep28-Pep35 series, in which the second tryptophan of the motif, Trp45, was substituted. Thus, aromatic residues with a preference for tryptophan are crucial for the motif. This conclusion was further corroborated by adding additional tryptophans, e.g., at position 40 (Pep11, or a phenylalanine in Pep5), position 46 (Pep41) or both (Pep42). Here, all peptides gave rise to an increased emission intensity reflecting increased affinities. Similar effects were also observed when additional negative charges were added to the motif (see, e.g., Pep8, Pep9, Pep20 and Pep25). Thus, a large overall negative charge is important, likely matching the overall positive surface of ubiquitin. Finally, introducing residues with a higher propensity for β-strand formation also enhanced emission and thus apparent affinity (Pep38, Pep39 and Pep40), whereas helix-promoting residues (Pep37) had no or negative effects on binding suggesting that the peptides are unlikely to form the classical helical structure when bound to ubiquitin (see Supplemental Table S1 for helix propensities of all peptides). Collectively, these data suggest that high negative charge, high hydrophobicity with a preference for aromatics, in particular tryptophan, and adopting an extended structure are preferred.

To investigate the binding specificity, we tested the peptides for interaction with the structurally related small ubiquitin-like modifier (SUMO), a ubiquitin-like protein also used as posttranslational modification, but involved in different signaling pathways [57]. SUMO binds to extended stretches of residues with an interaction motif consisting of *φφXφ*, often followed by negatively charged residues or phosphorylation sites, where *φ* represents hydrophobic residues [58]. Thus, known SUMO binding features partly overlap the features extracted above. Fluorescently labeled SUMO was added to the peptide array. As controls, two peptides with known SUMO interacting motifs, i.e., VIDL from protein inhibitor of activated STAT X (PIASX), binding SUMO with 1 μM affinity [59], and the VVVI from promyelocytic leukemia protein P (PML-P) with an estimated weaker SUMO affinity in the 100 μM range [60] were included in the array (Pep47 and Pep48, respectively). A similar dependency on aromatic and negatively charged residues on the apparent affinity was observed (Fig. 1). However, the range of fluorescent signals and therefore apparent affinities was generally smaller for SUMO compared to ubiquitin, suggesting that SUMO is a more promiscuous interaction partner. For the two controls, one showed high apparent affinity (Pep47, PIASX), whereas the other (Pep48, PML-P) showed a lower-than-average affinity, all in accordance with their relative affinities. None of the peptides displayed an emission intensity comparable to those measured for ubiquitin, suggesting overall much weaker affinities. Thus, the motif appears to bind preferentially to ubiquitin over SUMO (Fig. 1), a structurally related homologue. Nedd8 binding was not addressed.

Building on a shared feature of a three-residue separation between the two aromatic residues of the known ubiquitin-binding sites in Dss1 and Cdc34, all peptides tested so far were constrained to this separation. To address the effect of elongating (4X) or shortening (2X) this distance on ubiquitin binding, we designed two peptides from the wild-type Dss1 peptide GDDTLWENNWDDEDI, where we deleted or inserted an asparagine between the tryptophans, not to change the charged state of the peptide. From NMR titrations of ^*15*^ N-ubiquitin with these peptides the peptide with a separation of two residues bound ubiquitin and with similar low affinity as the wild-type Dss1 (*K*_*d*_ = 3 mM). The 4 × peptide-induced changes to the chemical shifts of the same resonances but reflecting a much weaker affinity that did not allow for extraction of a *K*_*d*_ (Fig. S2). Thus, the distance between the aromatic residues of the DisUBM is optimally two or three residues.

In conclusion, the peptide binding array results showed that several aromatic and negatively charged residues are important for ubiquitin binding. Furthermore, we observed a distinct preference for tryptophan over phenylalanine and tyrosine as the aromatic residues and with an optimal separation between the aromatic residues of two or three residues. As the ubiquitin-binding motif is presented in a fully disordered context, we term the motif a “Disordered Ubiquitin Binding Motif” (DisUBM). The proposed motif can be loosely defined as XΩXXXΩX, where Ω is an aromatic residue, and X is any residue with a strong bias toward negatively charged residues (Fig. 1).

### DisUBM is present in a large range of diverse proteins of the degradome and transcriptome

To investigate if DisUBMs exist in other disordered proteins, we performed a bioinformatics search targeting the DisUBM motif in disordered, charged regions. Since Cdc34 and Dss1 are best characterized from humans and the yeasts *S. cerevisiae* and *S. pombe* [36, 38, 61, 62], we limited our search to these species*.* For human and *S. cerevisiae*, we based our predictions on a previous set of disordered proteins [63], while *S. pombe* was manually processed with DisEMLBL [64] and IUPred [65]. For each proteome, a conservative regular expression-based motif search was employed for XXXXXX[F/Y/W]XXX[F/Y/W]XXXXXX, keeping the distance between the aromatics as 3X. All hits were processed using the ExPASy pI/mW and ProtParam tools [66] and the localCider program [67] to calculate pI values, hydropathy scores, and net charge per residue (NCPR) values, respectively. The list was subsequently filtered using a pI cutoff of < 5 matching the average negative pI of the yeast proteome [68], a net charge per residue cutoff of < − 0.2 to include the known candidates, and a disorder cutoff of > 50% to increase the reliability of the motif to be in a disordered region. This procedure led to 44, 34 and 12 hits for human, *S. pombe* and *S. cerevisiae*, respectively (Supplemental Table S2). The list showed a diversity of proteins, many with GO terms linked to transcription (e.g., the transcription factors Not3 and Not5 and co-regulators as LEO1 and MED1) or ubiquitin signaling and/or ubiquitin-dependent degradation. Of this latter group, we selected eight different proteins for further investigation (Table 1). In this set of proteins, we identified Spd1 from *S. pombe*, which is also an IDP, currently investigated in house [42, 69] (Table 1). To test the conclusions from the peptide array that at least two aromatic residues are needed, we also included the *S. cerevisiae* yeast protein, Cue3, which carries only one aromatic residue in the motif, and which is located downstream of a known, folded ubiquitin-binding domain, CUE [70]. For the selected proteins, we additionally observed that the DisUBMs were all located more than 30 residues distant to any folded domains or known binding UBMs (Fig. S3) and that the helix propensities of the motifs were generally lower than 1% (Supplemental Table S2), highly similar to the known DisUBMs (Table 2). Thus, the DisUBM can be investigated as short-independent peptides.

**Table 1:** Properties of potential DisUBM-carrying proteins identified from a bioinformatic search

Protein	Motif sequence	Function	pI^a^	NCPR^b^	Hydropathy (GRAVY)^c^	Uniprot
spDss1_35-52_	TGDDT**LWENNWD**DEDIG	Proteasome assembly, DNA repair, apoptosis	3.2	− 0.41	− 1.6	O14140
spSpd1_103-120_	DFEEP**EWLKPFD**VVMEG	Cell cycle regulation	3.7	− 0.29	− 0.6	Q10585
spPtr1_2013-2030_	SEQDD**EFQWEWN**TETPS	E3 ubiquitin ligase	3.3	− 0.35	− 2.1	O13834
spRpn2_951-966_	ASPPE**DFEYPFD**DDD	Regulatory subunit of proteasome	3.2	− 0.47	− 1.6	O74762
hUB2R2_201-218_	NSSDL**LYDDLYD**DDIDD	E2 ubiquitin-conjugating enzyme	3.1	− 0.47	− 1.2	Q712K3
hUB2R1_201-218_ (Cdc34)	EGSDL**FYDDYYE**DGEVE	E2 ubiquitin-conjugating enzyme	3.2	− 0.47	− 1.3	P49427
hASCC2_606-623_	PYHSV**YYEDEYD**DTYDG	DNA repair by ubiquitin interaction	3.7	− 0.32	− 1.8	Q9H1I8
scCue3_428-445_	EADED**ERDDTYD**EADVN	Ubiquitin binding by the CUE domain	3.5	− 0.52	− 2.2	P53137

^a^Calculated by the ExPASy pI/mW tool [66]

^b^Net charge per residue, calculated using localCider [67]

^c^Calculated by the ExPASy ProtParam tool [66]

**Table 2:** A α-Helix propensities in UIMs and DisUBMs

Predicted α-helix propensity calculated using Agadir		
UIMs		Protein/Species
SEEDMIEWAKRESEREEEQR	19.55	EPS15(P42566)/Human
QEQEDLELAIALSKSEISEA	20.16	EPS15(P42566)/Human
EEELQLQLALAMSKEEADQP	23.47	EPN1(Q9Y6I3)/Human

DisUBMs		
GDDTLWENNWDDEDI	0.32	Dss1/S. pombe
DDTLWWENNWWDDED	0.41	n.a
DLWENNWDLWENNWD	0.73	n.a
DDTLWWEDDWWDDED	0.15	n.a
DLWEDDWDLWEDDWD	1.06	n.a

To test if the motifs from the eight proteins extracted from the list interacted with ubiquitin, the sequences containing the DisUBM were purchased as 15-mer peptides, similarly in length to the peptides in the array. The peptides were added to ^15^N-labeled ubiquitin and the chemical shift changes followed in a series of ^15^N,^1^H- heteronuclear single quantum coherence (HSQC) NMR spectra. NMR chemical shifts of ubiquitin are available (BMRB entry 4769 [46]), and however, due to different conditions and sequence (including the additional cysteine), an incomplete overlap of the spectra was observed. Therefore, to enable information-extraction at residue-level resolution, the peaks in the ^15^N,^1^H-HSQC spectrum of ubiquitin were assigned from ^15^N,^1^H-HSQC-NOESY and ^1^H,^15^N-HSQC-TOCSY spectra resulting in assignment of 63 of the 73 possible peaks (76 when including two prolines and the N-terminus).

**Fig. 2:**
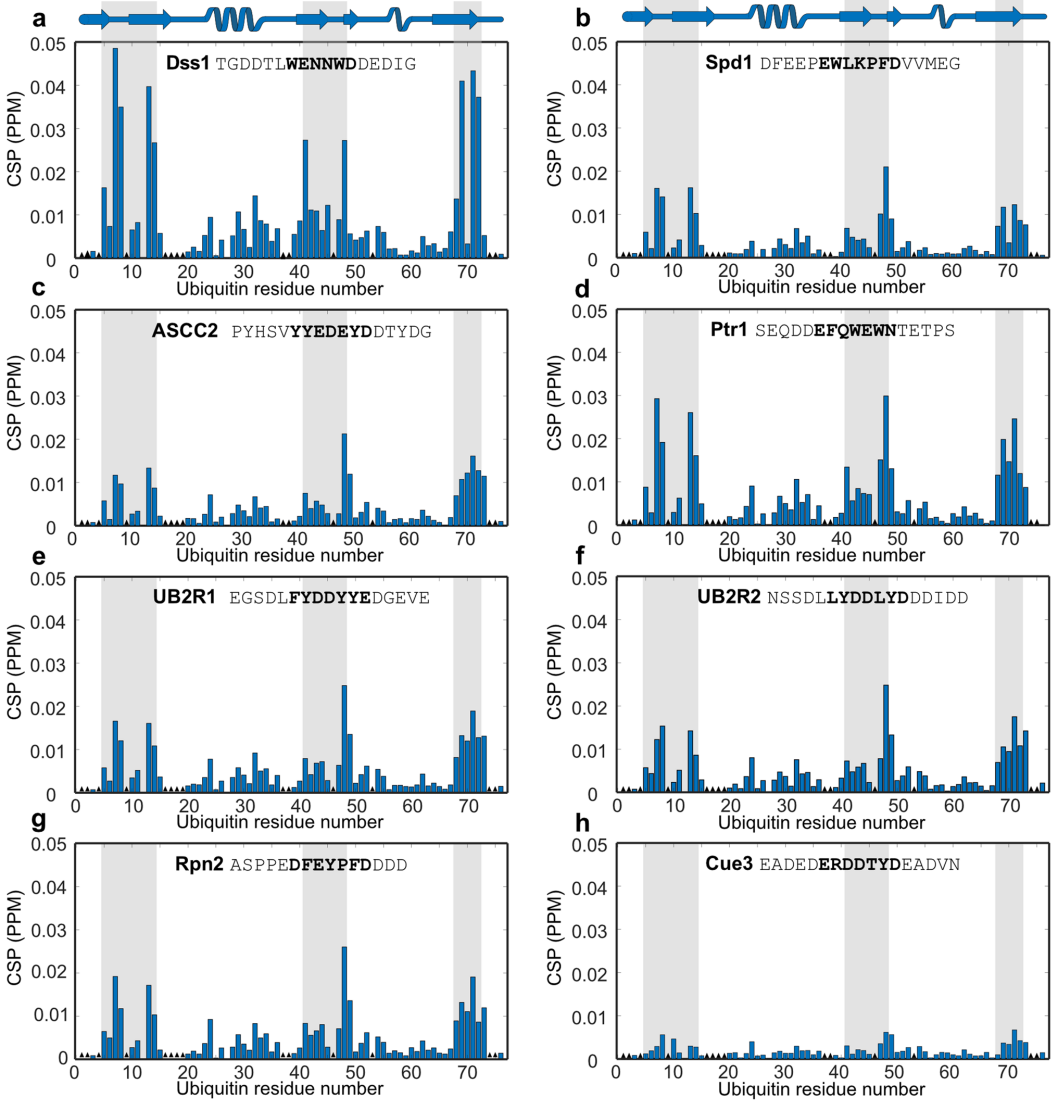
DisUBMs in different IDPs bind to the same patch on ubiquitin. a-h: CSP per residue of 100 µM ^15^N-labeled ubiquitin upon addition of 1 mM 15-mer peptides analyzed by ^1^H-^15^N-HSQC NMR. The secondary structure of ubiquitin is shown in the top cartoon and names and sequences of peptides indicated in each plot. The gray bars indicate the interaction site on ubiquitin as defined by the Dss1 peptide. The bolded part of the sequences indicates the proposed binding motif. The black triangles in the bar plot designate unassigned residues

All eight peptides were added separately to ^15^N-labeled ubiquitin at a ratio of 10:1. In all cases, except for the Cue3 peptide, they induced chemical shift perturbations (CSPs) in the HSQC spectrum of ubiquitin (Fig. 2). As previously observed for Dss1 and Cdc34 [36, 38], the DisUBMs bound to the canonical binding surface of ubiquitin centered around Ile13 and Leu69. Indeed, the patterns of the CSPs were very similar for all peptides, whereas the amplitudes of the perturbations clearly differed (Fig. 2). The UBS1 from Dss1 stands out by inducing much larger CSPs (Fig. 2, correlation plots in Fig. S4), while for Cue3, which carries only one aromatic residue in the motif, but similar overall negative charge, only very small CSPs were apparent, further underlining the importance of the pair of aromatic residues in the DisUBM (Fig. 2h). We then attempted to determine the affinity of the peptides from Spd1 and Dss1 by following the chemical shifts through a titration. From a global fit of the CSPs of residues for which the CSPs were larger than the average plus one standard deviation, the *K*_*d*_s were determined to be 3100 ± 400 µM and 3000 ± 200 µM for Spd1 and Dss1, respectively (Fig. S5b). These numbers imply, as expected, that the amplitudes of the CSPs not necessarily are direct measures of the affinity. The presence of tryptophans with high electron density and the difference in the number of negative charges clearly impact the amplitudes of CSPs of ubiquitin, across the peptides. Finally, to address if the DisUBM is solely relevant in proteins with functions in ubiquitin signaling, we selected two proteins with roles in transcription for which we have identified a potential DisUBM and tested if the corresponding peptides would bind ubiquitin. The two peptides represented the DisUBMs from human zinc finger and BTB domain-containing protein 1 (ZFB) and RNA polymerase II-associated protein 1 from *S. pombe* (POLIIBP). Both peptides had similar pI and helicity but varied in the types of aromatic residues. Both peptides induced chemical shift perturbations of the same residues on ubiquitin, although these were small and especially the peptide from ZFB, which carries a phenylalanines bound very weakly (Fig. S5d, e), as also expected from the results of PepSpot assay. Thus, since the data were recorded at a maximum peptide concentration of 1 mM, these data highlight the functionally broad distribution of the DisUBM, but also again show that tryptophan plays a larger role compared to the other aromatic sidechains.

In conclusion, the DisUBM is present in a variety of proteins spanning a versatile functional range. The motif binds to the canonical binding site on ubiquitin with variations in affinity depending on the specific sequence of the peptides. Two or more aromatic residues are required for reaching a relevant affinity.

### Binding to ubiquitin shows context-dependent behavior

**Fig. 3:**
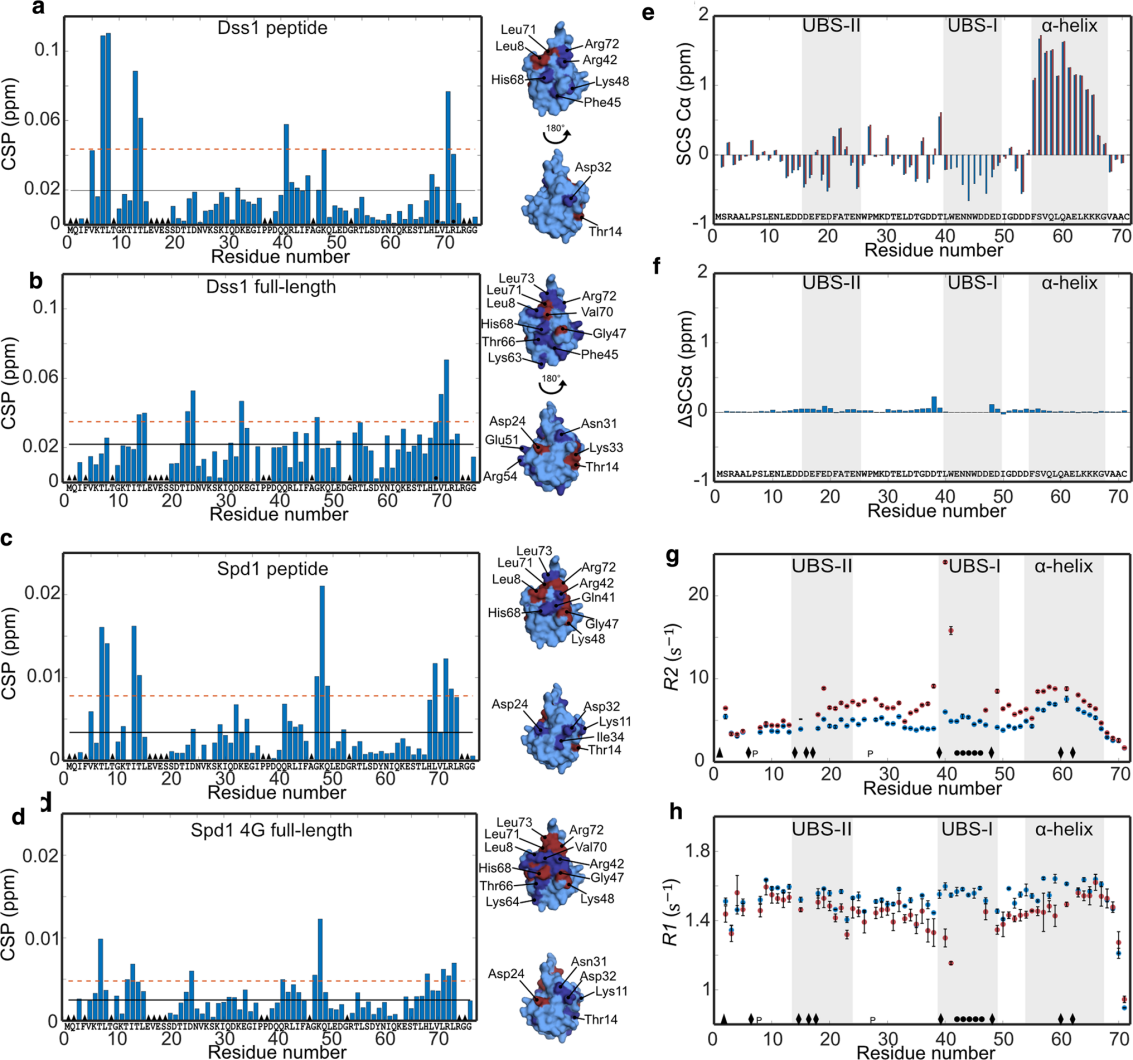
The context of the DisUBM affects binding. **a** CSP per residue of 100 µM ^15^N-labeled ubiquitin added 3000 µM Dss1 peptide. The black line indicates the average CSP and the dashed red line the average CSP + one standard deviation. Black triangles indicate unassigned residues, and black circles line broadening upon addition of Dss1. Surface representations of ubiquitin with affected residues in dark blue (above average CSP) and red (above average + one standard deviation) highlighted are shown to the right. **b** Same as **a** but with 440 µM full-length Dss1. **c** Same as **a** but with 1000 µM Spd1 peptide. **d** Same as **a** but with 290 µM 4G Spd1. **e** SCS of the C^α^ atoms of 1 mM ^13^C-^15^ N-labeled Dss1 alone (blue) or with 2 mM ubiquitin (red). Gray bars indicate the transient helix and ubiquitin-binding sites in Dss1 as identified by Paraskevopoulos et al. [36]. **f** Differences between the SCSs shown in **e** of Dss1 with and without ubiquitin. **g** *R*_*2*_ and **h** *R*_*1*_ values of 500 µM ^15^ N-labeled Dss1 without (blue) and with (red) 1 mM ubiquitin. The error bars are standard deviations from triplicate recordings on the same sample. Triangles are unassigned residues, diamonds residues with peak overlap, “*p*” indicates prolines and circles peaks that disappear upon addition of ubiquitin

In some cases, a SLiM alone may not represent the entire binding event, and the context of the disordered chain can impact the interaction both positively and negatively [25, 71]. To address if the chain context of the DisUBM would have any effects on binding affinity we used full-length proteins of Dss1 and Spd1. We first recorded a series of HSQC spectra on ^15^N-labeled ubiquitin with increasing concentrations of full-length Dss1 and compared these to those of the peptides. To measure the affinity of the complex, titrations were again performed. For Dss1, the *K*_*d*_ was 380 ± 40 µM, almost 10 times lower than for the peptide alone. For Spd1, we mapped the interaction on the full-length protein using ^1^N-labeled Spd1 (Fig. S5a). Due to the low solubility of Spd1, the interaction was mapped on a 30 µM ^15^N-Spd1 sample added 560 µM ubiquitin. Although the CSPs were very small, distinct effects were seen in the DisUBM, suggesting this specifically engages in the interaction, leaving the remainder of the chain affected to a much lesser extent. To increase solubility of Spd1, we made two different variants: In Spd1-4G, we mutated four large hydrophobic residues distant to the DisUBM with glycine and in Spd1-CTD, we deleted the hydrophobic N-terminal 49 residues. These variants were added to ^15^N-ubiquitin (Fig. 3d and Fig. S5e). Here, and similar to Dss1, we observed smaller amplitudes of the CSPs and more distributed effects. Thus, comparing the peptide interactions with the full-length interactions revealed context effects. On a residue basis, the CSPs observed in ubiquitin upon addition of Dss1 suggested that the interaction of full-length Dss1 engages a much larger surface of ubiquitin compared to that of the peptide (Fig. 3, left), which also explains the increased affinity. For Spd1, this was less pronounced and in line with this, we were unable to reach saturation at the workable concentration, suggesting that context plays a lesser role for Spd1 (Fig. 3). This also corroborates that the DisUBM in Spd1 is the main site for the interaction, and thus that the affinity for ubiquitin is likely more similar for the peptide and the full-length protein.

In conclusion, the study of two different proteins containing the DisUBM suggests that the affinity of the DisUBM can be dependent on the local and global sequence and thus the context of the host protein. This would allow for fine-tuning of affinities not only by variation in the motif sequence itself, but also through effects imposed by the remainder of the disordered protein chain.

### DisUBM likely remains disordered when bound

To assess the structure of the bound DisUBM, we first predicted the α-helix propensity of the different DisUBM sequences using Agadir, a webtool that predicts the propensity based on experimental data and modeling of the energy function of individual residues [72]. For classical UIMs, Agadir predicted significant α-helix propensity, as expected, whereas for the DisUBMs only negligible propensity for α-helix was seen (Table 2). This suggests that the DisUBM may rely on different structures than helix formation for its interactions with ubiquitin. To shed light on this, we performed a reverse titration of unlabeled ubiquitin added to ^15^N,^13^C-labeled Dss1. In accordance with earlier studies, the signals from residues in the DisUBM in Dss1 (UBS1) disappeared upon addition of ubiquitin, both in ^15^N-HSQC as shown previously [36], but also in ^13^C-filtered HSQCs and CON NMR spectra, making the structure of the bound DisUBM in the complex inaccessible. Nonetheless, secondary chemical shifts values of the C^α^-atoms close to the DisUBM and in the remainder parts of Dss1 did not reveal any major differences between the bound and free forms (Fig. 3f), and if anything, the binding region appeared extended in the complex. This supports results from the peptide array, where it was clear that helix-promoting substitutions were unfavorable, whereas residues promoting extended structure were favorable for ubiquitin binding. To finally address if structure formation would lead to changes in the dynamics of the chain we recorded longitudinal *R*_*1*_ and transverse *R*_*2*_ relaxation rates in the free and bound states of full-length Dss1 (Fig. 3g, h). Here, changes in both *R*_*1*_ and *R*_*2*_ close to the UBS1 were observed, supporting that the major contact site remains the UBS1, but changes in *R*_*2*_ were also observed throughout the chain, indicating the formation of a dynamic complex with multiple contacts along the Dss1 chain.

Thus, the DisUBM does not have inherent propensity for α-helix formation and likely remains extended and disordered when in complex with ubiquitin. The disordered chain constituting the DisUBM context dynamically contact the surface of ubiquitin and presumably through these contacts contribute to the affinity.

### Optimized affinity at the cost of context

The results from the peptide binding array and NMR analyses suggested that the affinity of the DisUBM from Dss1 could be increased, but also that the affinity relied on context. A key conclusion was that additional tryptophans and acidic residues within the 15-residue context enhanced affinity, especially when included in the core motif, which we define as WENNW in Dss1. Pep42 and Pep43 had the highest emission intensity in the peptide array and were therefore chosen for further investigation in a new set of peptides. Pep42 had two extra tryptophans and was thus called double tryptophan, PepDW for short. Pep43 had the core motif of WENNW repeated and was thus called double motif, PepDM for short. PepDW and PepDM as well as two additional peptides built on the Pep42 and Pep43 scaffolds, where the two asparagines of the core were substituted with aspartates (PepDWD and PepDMD) were purchased.

**Fig. 4:**
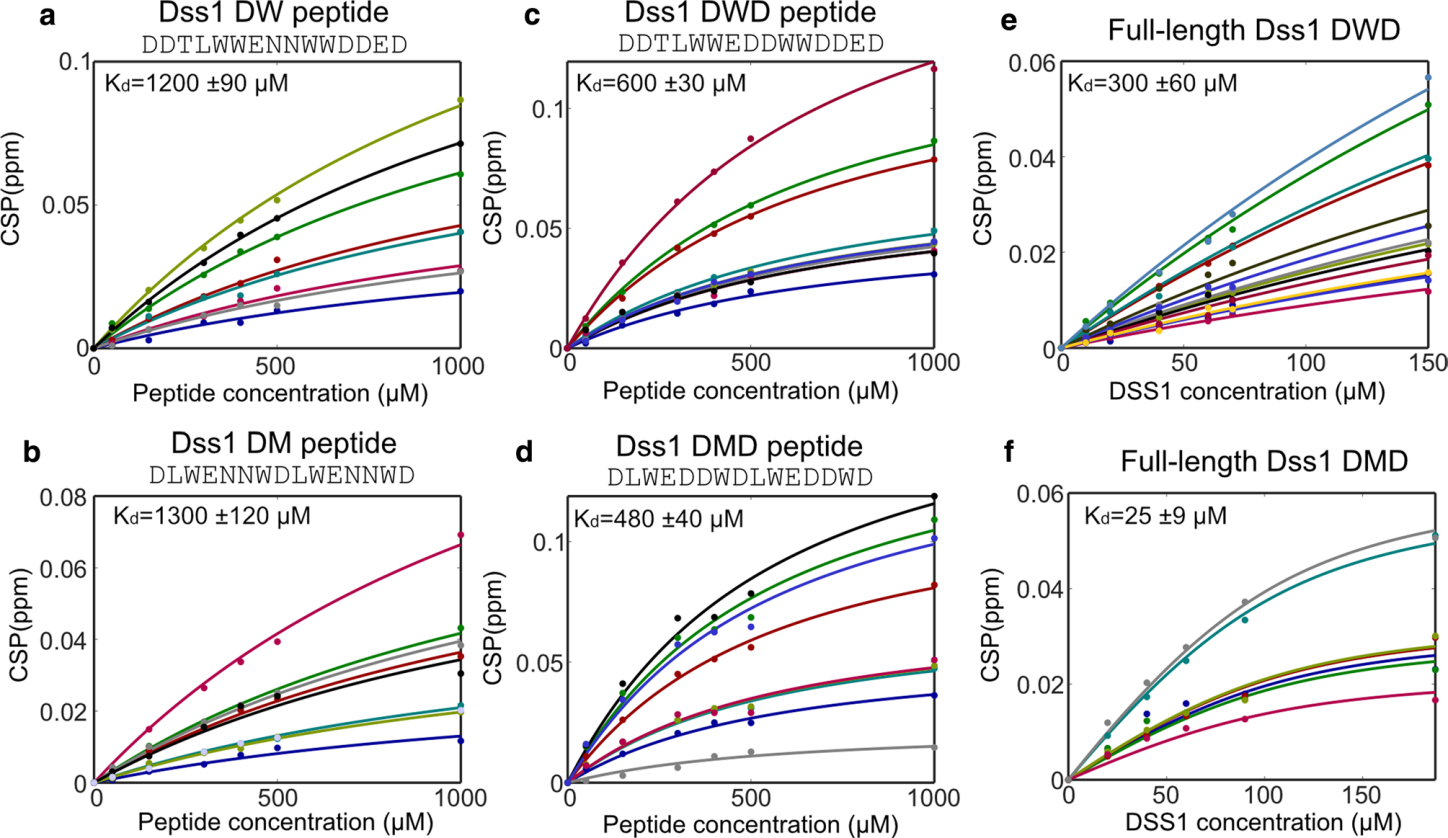
Increased affinity for ubiquitin by aromatics, negative charge and chain context. Changes in CSP with the concentrations of peptide or protein. Each fit line and corresponding colored data points reflect the CSPs of individual NMR signals from ^1^H-^15^ N-HSQC spectra of 100 µM ^15^ N-labeled ubiquitin upon titration with either peptides (**a**–**d**) or proteins (**e** and **f**). Peptide and protein names are shown at the top. All data points in one titration were fitted using a global fit to obtain the *K*_*d*_ values reported on each plot

Titration of ^15^ N-labeled ubiquitin with the new set of four peptides was followed by NMR and revealed that the affinity of the motif with additional tryptophans increased twofold compared to the WT *K*_*d*_ of 3000 µM to *K*_*d*_*s* of 1200 ± 90 µM and 1300 ± 120 µM for the PepDW and PepDM, respectively (Fig. 4a, b). With the additional aspartic acids in the core motif, the affinity increased further to *K*_*d*_s of 600 ± 30 µM (PepDWD) and 480 ± 40 µM (PepDMD), respectively (Fig. 4c, d). Thus, the motif inherently has a low affinity, which can be substantially increased by a few substitutions. Since the context of the full-length Dss1 had large effects on the interaction with ubiquitin, we subsequently addressed if this was also true for full-length Dss1 and inserted DWD and DMD sequences into it. In these cases, the affinity for ubiquitin was 300 ± 60 µM for full-length DWD, and hence not significantly different to that of the peptide alone, and 25 ± 9 µM for full-length DMD, higher affinity than for the peptide alone, suggesting context effects (Fig. 4e, f).

**Fig. 5:**
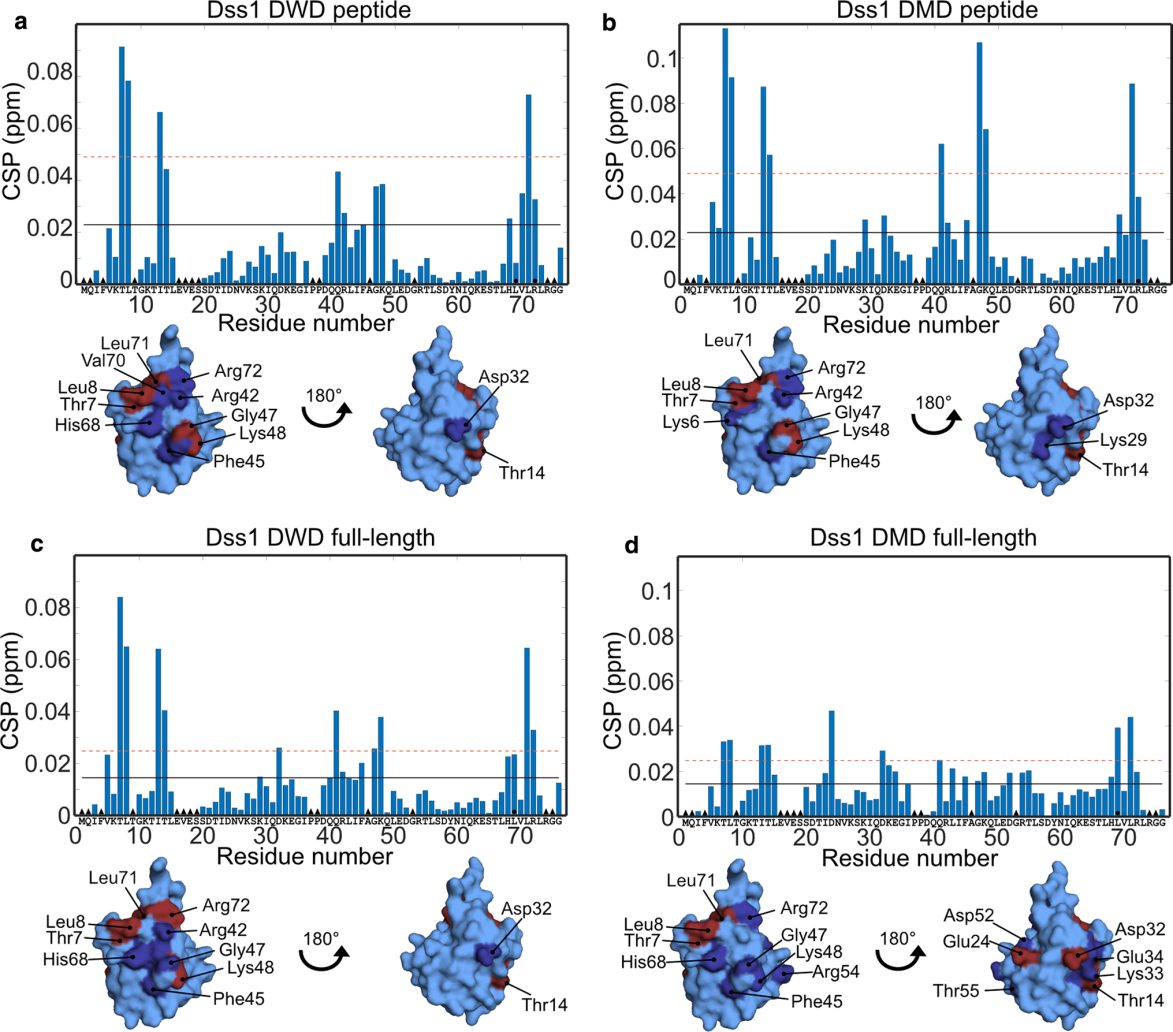
Binding surfaces on ubiquitin of optimized Dss1 variants. CSP per residue of 100 µM ^15^ N-labeled ubiquitin with **a** 300 µM DWD peptide, **b** 500 µM DMD peptide, **c** 150 µM full-length DWD Dss1, **d** 90 µM full-length DMD Dss1. Residues with CSP values above average and above average plus one standard deviation are shown on the surface of ubiquitin in dark blue and red, respectively. Black lines indicate mean CSP value, the red dashed line the mean + one standard deviation. The black triangles indicate unassigned residues, black circles line broadening or peak coalescence

Analyses of the CSP profiles of ^15^N-labeled ubiquitin titrated with the peptides PepDWD and PepDMD showed that the peptides bound to the canonical interaction surface of ubiquitin (Fig. 5). This is similar to what was observed for the naturally occurring DisUBM peptides (Fig. 2). Full-length Dss1-DWD also bound to this surface, but in this case, it did so without engaging the additional contacts within the context, contrary to what was observed for WT Dss1. For Dss1-DMD, and similar to Dss1-WT, context effects were evident, providing an increase in affinity. The difference in affinity gain for Dss1-DWD (twofold compared to PepDWD) and Dss1-DMD proteins (20-fold compared to PepDMD) could thus be explained by the difference in context behavior (Fig. 5). However, having a double motif also increases the probability for rebinding through a local concentration effect, which thereby contributes to an avidity effect.

### The Dss1 variants are functional in cells

**Fig. 6:**
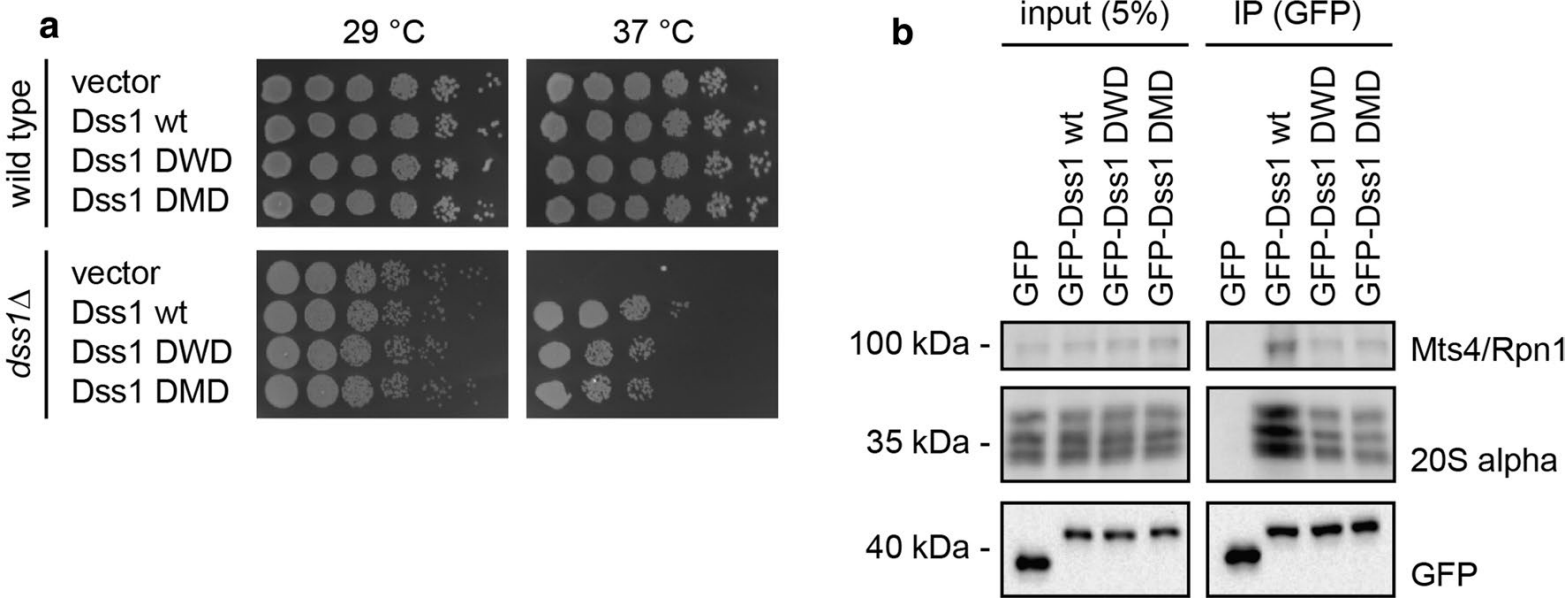
Cellular effects of DisUBM variants. **a** Yeast growth assays with wild-type and *dss1*Δ *S. pombe* cells. Serial fivefold dilutions of the indicated strains were spotted in droplets onto solid media and incubated at 29 °C or 37 °C. **b** Yeast cells expressing GFP and the indicated GFP-tagged Dss1 variants were lysed and used for immunoprecipitation (IP) assays using GFP-trap resin. Samples of the input and the precipitated material were analyzed by SDS-PAGE and western blotting using antibodies to the 19S proteasome subunit Mts4/Rpn1, the 20S proteasome α-subunits, and GFP

To investigate how the Dss1 variants with increased affinity for ubiquitin might affect the fitness of cells, full-length DWD and DMD were introduced in *S. pombe* cells either with or without the endogenous *dss1*^+^ gene. We observed that Dss1-DWD and Dss1-DMD had no significant effect on the growth of wild-type *S. pombe* cells suggesting that the increased affinity for ubiquitin does not lead to dramatic cytotoxic effects (Fig. 6a). Expression of the Dss1 variants rescued the temperature-sensitive growth defect of a *dss1*Δ mutant, albeit at a slightly reduced level compared to wild-type Dss1 (Fig. 6a). Since most of the *dss1*Δ temperature-sensitive phenotype seems to be derived from lack of Dss1 in the 26S proteasome [36, 54], this suggests that the Dss1 variants are largely functional and incorporated into 26S proteasomes. Indeed, this appeared to be the case, since GFP-tagged versions of the Dss1 variants efficiently co-precipitated 26S proteasomes, albeit the Dss1-DWD and Dss1-DMD variants appeared to interact slightly less efficiently than wild-type Dss1 (Fig. 6b). In conclusion, this indicates that the increased affinity of the Dss1-DWD and Dss1-DMD variants comes at the cost of a slightly reduced affinity for the proteasome, and the increased affinity for ubiquitin is not sufficient to cause any global perturbations to the UPS and concomitant toxic effects.

### DisUBM emerges from screening of unbiased cyclic peptide libraries

**Fig. 7:**
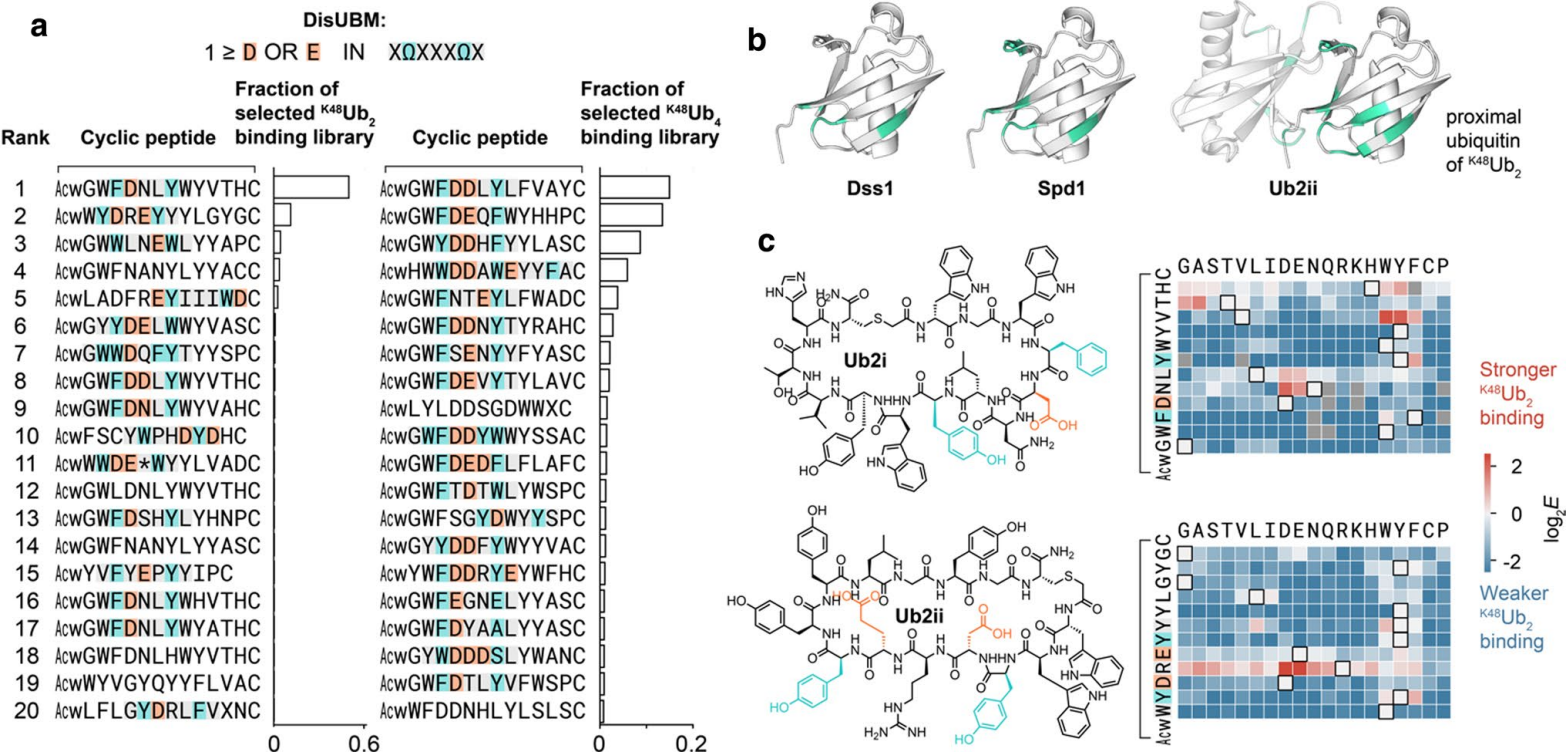
DisUBMs feature in de novo cyclic peptides from unbiased screens. **a** De novo cyclic peptides from trillion-scale random cyclic peptide library are enriched in DisUBMs. Shown are the 20 highest-ranked cyclic peptides after 4 rounds of RaPID selection against ^K48^Ub_2_ or ^K48^Ub_4_. Cyclic peptides containing DisUBMs have motif highlighted: XΩXXXΩX, where Ω is F/Y/W, X is any amino acid, and motif contains at least one E/D. Figure adapted from [45]. **b** Binding of natural DisUBM and de novo cyclic peptide induce similar chemical shift perturbations (CSP) in monomeric and proximal ubiquitin of ^K48^Ub_2_, respectively. CSP one standard deviation above the mean for Dss1 and Spd1, and CSP > 0.3 for ^K48^Ub_2_ [45] are shown in cyan (structures based on pdb 1aar). **c** Deep mutational scanning of cyclic peptides **Ub2i** and **Ub2ii** binding to ^K48^Ub_2_ reveals that mutations which increase DisUBM character improve binding. In particular, mutation to D/E between the two aromatic amino acids of DisUBM ΩXXXΩ

Given the small size of DisUBM, this motif could plausibly appear in peptides arising de novo from screens of randomized sequence libraries. Fortunately, Nawatha et. al. recently conducted such a screen. Using the RaPID system [73], a trillion unique cyclic peptides, with 8–12 randomized amino acids per peptide, were screened against chemically synthesized K48-linked ubiquitin dimers (^K48^Ub_2_) and tetramers (^K48^Ub_4_) [45]. After four rounds of selection, de novo cyclic peptide binders for ^K48^Ub_2_ and ^K48^Ub_4_ were found, with affinities in the low nM *K*_d_ range and selectivity for chain length and linkage-type. While the DisUBM motif, XΩXXXΩX with at least one negative amino acid, was moderately abundant in the initial library (estimated to be 1–2% of sequences), the top-ranked de novo cyclic peptides were markedly enriched in DisUBMs (Fig. 7a): 75% and 90% of the top twenty sequences from the ^K48^Ub_2_ and ^K48^Ub_4_ screens, respectively. Furthermore, the reported CSPs in the proximal ubiquitin of ^K48^Ub_2_ induced by one of the hit peptides Ub2ii are similar to the CSPs induced by Dss1 and Spd1 in monomeric ubiquitin (Fig. 7b), suggesting a shared binding site for the natural and de novo DisUBMs. We note that the de novo peptides had both two and three residues separation between the aromatic sidechains with the top-ranked peptides dominated by a separation of three.

To explore the sequence determinants of the DisUBM in these de novo cyclic peptides, we conducted deep mutational scanning for four of the hits [44]. Saturation mutagenesis libraries, including mutations to canonical and non-canonical amino acids, were pooled and their relative binding to ^K48^Ub_2_ or ^K48^Ub_4_ assessed through the calculation of enrichment ratios (Fig. S6, Supplemental note). Interestingly, mutations that are beneficial for binding are those that introduce negatively charged or aromatic amino acids (Fig. S6, Fig. 7c), suggesting that increasing the DisUBM character via increased negative charge and aromaticity can increase the affinity for ubiquitin chains. However, unlike the natural IDP DisUBM, mutations introducing negative charges were only tolerated between the two critical aromatics of the motif. Mutations outside the two aromatic residues may interfere with the sequence features that allow these cyclic peptides to interact with high-affinity nM *K*_d_ and discriminate K48-linked ubiquitin chains.

## Discussion

**Fig. 8:**
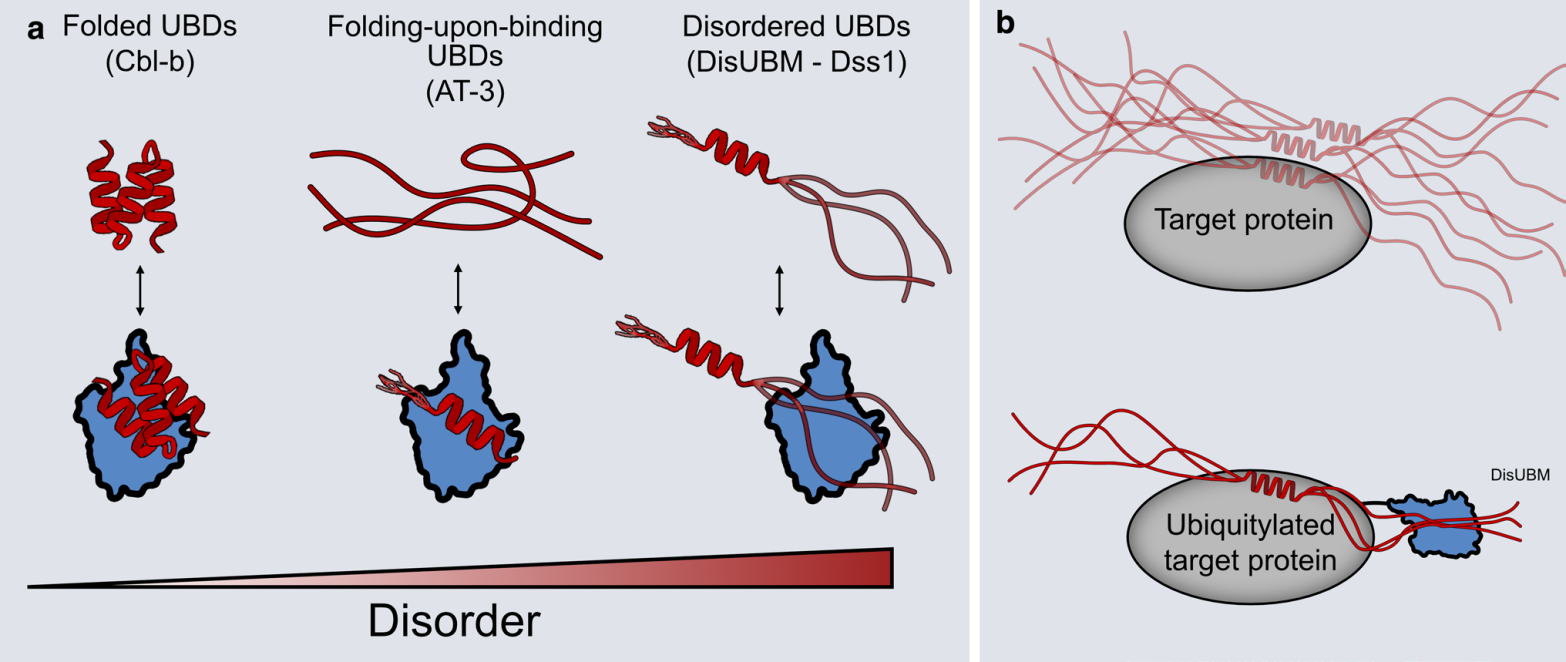
Disorder in ubiquitin binding. **a** Canonical UBDs (red) are known to have stable secondary or tertiary structures both in isolation and when binding ubiquitin (blue) (left). Recently, it was shown that some UBDs only fold upon binding to ubiquitin (middle). This study has revealed that a ubiquitin-binding motif, DisUBM occurs frequently in IDPs and that this binds ubiquitin without adopting any fixed secondary structure (right). **b** The DisUBM motif is suggested to act as an affinity enhancer when IDPs bind to ubiquitylated folded binding partners. The folded binding partner could be ubiquitin itself

Ubiquitin-binding modules exist in many different proteins carrying out highly versatile tasks ranging from protein degradation to signal transduction and regulation [14, 74, 75]. For years, these modules have been seen as requiring a folded structure to interact with ubiquitin, either in the form of a small, folded domain or a single α-helix, but recent computational studies have suggested that some modules may retain some dynamics and disorder in the free state, allowing for folding-upon-binding to ubiquitin to canonical helical structures [35]. In the present work, we extend the spectrum of ubiquitin-binding modules to account for disordered extended structures in complex with ubiquitin. We have coined this module the disordered ubiquitin-binding motif, DisUBM (Fig. 8a).

Using a peptide array, we decomposed the determinants for disordered ubiquitin binding and discovered a strict need for having at least two aromatic residues, preferably tryptophans, and several negative charges. The importance of the distance between aromatic residues was not defined, and however, the screen of randomized cyclic peptides indicates that a separation by three residues is optimal. Bioinformatics searches in a disordered protein database revealed many proteins with known or predicted functions in the UPS and in transcription carrying potential DisUBMs, and seven of these were confirmed to bind ubiquitin in vitro directly exploiting their predicted DisUBM. However, the sequence conservation within DisUBM is low, and the DisUBMs alone did not carry much interaction energy, suggesting an overall inherent low affinity by the motif. There are several possible explanations for these observations. First, DisUBMs are located in disordered chains, which themselves carry several SLiMs and interaction sites of relevance to their function, and thus, binding ubiquitin might not be their main task. Ubiquitylation is a posttranslational modification, and once a binding partner for an IDP becomes ubiquitylated, it is possible that the DisUBM acts to enhance the affinity to an already recognized target and hence works in concert with the PTM (Fig. 8b). For Dss1 and Spd1, known ubiquitylated partners are BRCA2 [76] and PCNA [77], respectively, highlighting the possibility for such a mechanism, and also explaining the low affinity of the isolated motif. This mechanism would also be consistent with the possibility of having DisUBMs located in disordered loops of otherwise globular proteins. We note that in our unbiased search for DisUBMs, we identified Cdc34 with a known DisUBM located in its disordered chain [38], and however, this disordered tail and hence the presence of the DisUBM is not conserved across a set of E2s [78]. Intriguingly, those E2s that lack the tail (UBE2G1 and UBE2G2) instead contain a DisUBM within an acidic disordered loop in the globular domain (although UBE2G2 only has one aromatic side chain) and this loop has through non-covalent ubiquitin interactions been shown to regulate the activity of these enzymes [79, 80]. Second, IDPs are known to carry overlapping SLiMs, which, together with an in-built tuning effect of another interaction may explain the high sequence divergence where only conservation of features matter. Finally, as the list of potential carriers of DisUBMs extracted in the present work was based on the Dss1 sequence as origin (Supplemental Table S2), the number of IDPs carrying a DisUBM is likely to be much larger.

In contrast to the well-described group of UBDs, the DisUBM showed no sign of helix formation upon binding. From the results of the peptide binding array, higher β-strand propensity (Pep38 and Pep39) increased ubiquitin affinity, whereas helix promotion (Pep36) reduced binding, indicating that an extended conformation is favorable for complex formation. This was to some extent corroborated by the secondary chemical shift analysis, although we were unable to capture the exact structural properties of the bound DisUBM and by the high negative charge promoting extendedness. Additionally, from a comparison of the motifs alone and in context of two different and unrelated proteins, Dss1 and Spd1, we observed that binding can depend very much on context. For Dss1, a large region of the chain engaged in wrapping of ubiquitin, which enhanced the affinity tenfold, and distributed the chemical shift changes across the ubiquitin surface. This type of complex formation has been observed for different IDPs, where such dynamic complexes have been termed “wrapping” [81] or “shuffle” [82] complexes, referring to the original description of dynamic complexes in enzymes as a hot potato binding mechanism [83]. For Spd1, context effects were much less evident, suggesting that increased affinity may be achieved from binding to other SLiMs in Spd1 targeting ubiquitylated binding partner. We were able to increase the affinity of the DisUBM of Dss1 by increasing the number of tryptophans and negative charges, whereby the affinity was increased to the low micromolar range. However, for Dss1-DWD, this came with a cost of context behavior, whereas for the strongest binding Dss1-DMD, context effects were preserved. The two variants differ in one particular way: For Dss1-DWD, there is just one DisUBM (WWEDDWWDD), whereas Dss1-DMD carries two separated DisUBMs (DLWEDDWD and DLWEDDWD). Higher affinity likely stems from two different mechanisms. For Dss1-DWD, increasing the lifetime of the bound state of the DisUBM, compromises the adaptation of the disordered chain to the surface of ubiquitin and the corresponding loss of context effects. For Dss1-DMD, on the other hand, fast exchange in the binding site allows for exchange between binding each of the two motifs, which not only enhances the affinity by increasing the local concentration through allovalent effects [84, 85, 86], but also allows the chain to continuously adapt to the ubiquitin surface adding affinity to the complex through context effects.

We did not observe a change in fitness of the cells by introducing the high-affinity DisUBM into Dss1. This suggests that the Dss1 variants are still functional, and indeed, they are incorporated into 26S proteasomes. Since we did not observe any toxic effect of expressing the high-affinity DisUBM variants in cells, the affinity, albeit increased, is likely not sufficiently high to cause any global perturbations of the UPS in the cells. Importantly, previous studies have shown that the UBS1 region in Dss1 is involved in both proteasome binding and assembly [62, 87], and in ubiquitin binding [36]. As the aromatic residues appear to be involved in both processes and their mutation causes a temperature-sensitive phenotype [36], it is not straightforward to separate these functions by mutation and we can therefore not directly test the cellular consequences of the ubiquitin-binding properties of Dss1. Moreover, notably, Dss1 plays multiple roles in the cell and has a large and diverse interactome [54, 62, 88, 89], so we cannot exclude that other Dss1 functions that we did not test for here are affected by the manipulations of the Dss1 DisUBM.

Recent screens of trillion-member randomized libraries found de novo cyclic peptides capable of binding ubiquitin chains, with subtle discrimination between chain lengths and linkage-type. The aromaticity and negative charge inherent to the DisUBM, discovered from two non-related IDPs, were recapitulated in the top-ranked peptides from these screens. Thus, the motif contains features broadly relevant for peptide ubiquitin interaction. Indeed, they are the preferred sequences and rise to the top of hits when screening unbiased random libraries.

The selectivity toward di-ubiquitin seen for the cyclic peptides may also be relevant to natural occurring DisUBM positioned in tandem. Indeed, in a previous study of Dss1, which carry two DisUBM pull-down assays showed a clear preference for ubiquitin chains [36]. With the suggestion made here that DisUBM may serve as an affinity enhancer, this may also be relevant in the case of modification with di-ubiquitin chains, when two or more tandemly positioned DisUBMs exist, or when a combination of one DisUBM with a canonical UBD is present in the protein (Fig. 8b).

## Conclusion

Modification by ubiquitin occurs for many different proteins carrying out hugely diverse cellular tasks. Correspondingly, numerous ubiquitin recognition and binding modules exist, typically forming helices prior to or after binding to ubiquitin. The present work expands the repertoire for ubiquitin recognition defining a disordered ubiquitin-binding motif, DisUBM, carrying features of at least two aromatic residues, with a preference for tryptophan, and several negative charges. The motif stays extended and disordered in complex with ubiquitin and can explore its disordered chain context to engage additional surface of ubiquitin to enhance affinity. The affinity of the DisUBM is inherently low, and we propose that the DisUBM acts as an affinity enhancer for disordered proteins when they bind to folded partners that can be regulatorily ubiquitylated in posttranslational events.

## Supplementary Information

Below is the link to the electronic supplementary material.

Supplementary file1 (PDF 3588 KB)

Supplementary file2 (XLSX 35 KB)

## Data Availability

All datasets generated by this study are included as tables in supplementary information.
