# Supplementary material for: A context-dependent and disordered ubiquitin-binding motif
Source: Cell Mol Life Sci. 2022 Aug 16;79(9):484. doi: 10.1007/s00018-022-04486-w (PMC9381478; doi:10.1007/s00018-022-04486-w)
Supplement: Supplementary file 1 — Supplementary file1 (PDF 3588 KB) [file 18_2022_4486_MOESM1_ESM.pdf]

SUPPLEMENTAL DATA

A context dependent and disordered ubiquitin binding motif

Jesper E. Dreier[1,2], Andreas Prestel[1], Joao M. Martin[3], Sebastian S. Brøndum[1], Olaf Nielsen[4], Anna E. Garbers[1,2], Hiroaki Suga[5], Wouter Boomsma[3], Joseph M. Rogers[6], Rasmus Hartmann-Petersen[2,7#], Birthe B. Kragelund[1,2,7#]

[1]Structural Biology and NMR Laboratory, [2]REPIN, [4]Functional Genomics, and [7]The Linderstrøm Lang Centre for Protein Science, Department of Biology, University of Copenhagen, Ole Maaloes Vej 5, DK-2200 Copenhagen N, Denmark; [3]Department of Computer Science, University of Copenhagen, Universitetsparken 1, DK-2100 Copenhagen Ø, Denmark; [5]Department of Chemistry, Graduate School of Science, The University of Tokyo, Tokyo 113-0033, Japan; [6]Department of Drug Design and Pharmacology, University of Copenhagen, Jagtvej 160, DK-2100 Copenhagen Ø, Denmark.

Contents

Supplemental Fig. S1. Raw data and controls of membrane bound peptide arrays
Supplemental Fig. S1. NMR data from interaction with aromatic distance variation
Supplemental Fig. S3. Disorder predictions of proteins containing the DisUBM
Supplemental Fig. S4. CSP Correlations of DisUBM peptides
Supplemental Fig. S5. NMR data from the interaction between ubiquitin, Dss1 and Spd1
Supplemental Fig. S6. Deep mutational scanning of ubiquitin chain binding *de novo* cyclic peptides.
Supplemental Note. Supplemental discussion of deep mutational scanning of ubiquitin chain binding *de novo* cyclic peptides.

Supplemental Table S1. Agadir helix propensities for peptides used in the Pepspot analysis.
Supplemental Table S2. List of proteins with potential DisUBM (separate excel file)

Supplemental References.

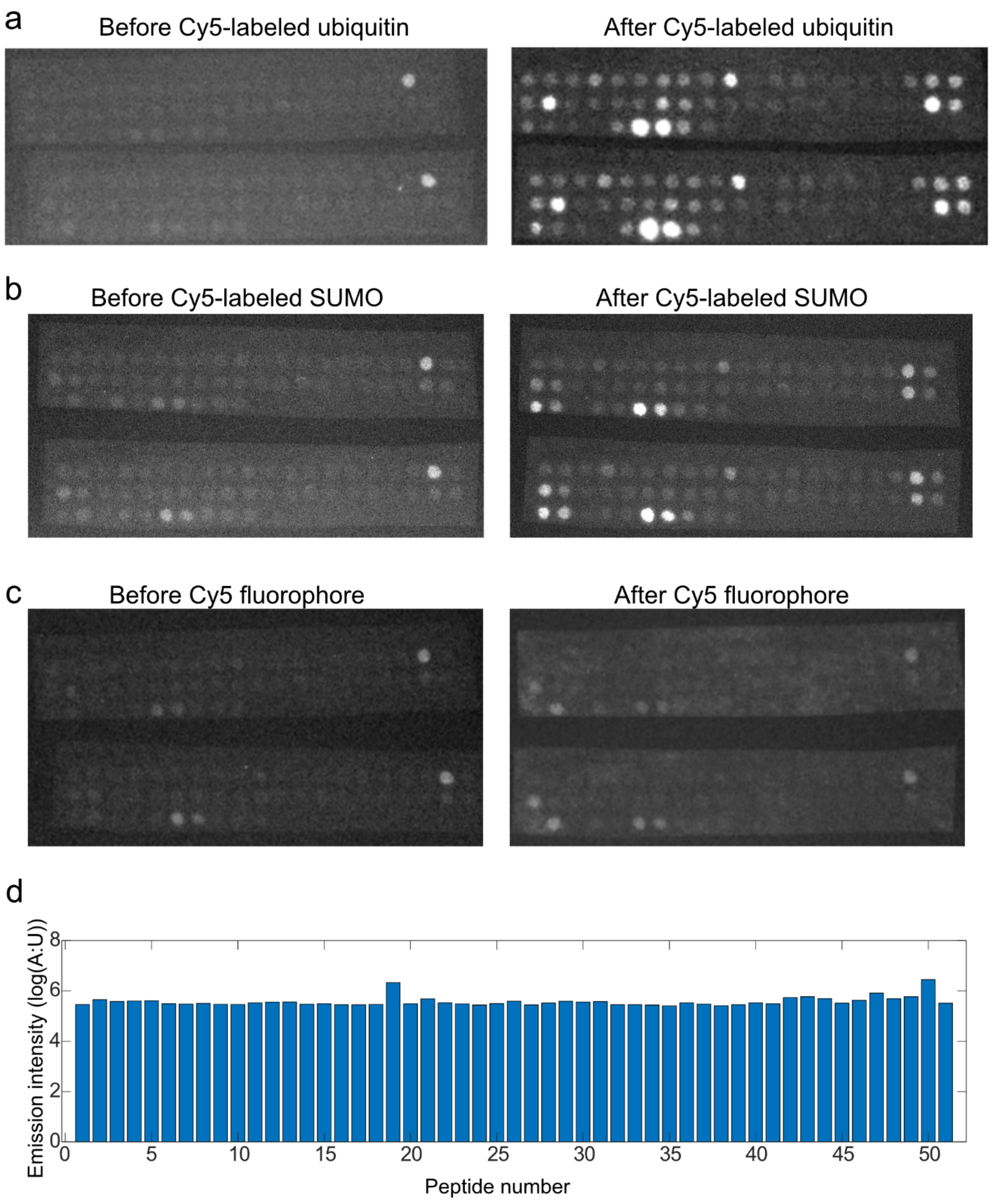

Fig. S1. Raw data and controls of membrane bound peptide arrays

a Pictures of the peptide arrays before and after incubation with Cy-5 labeled ubiquitin. **b** Pictures of the peptide arrays before and after incubation with Cy-5 labeled SUMO. **c** Pictures of the peptide arrays before and after incubation with Cy-5 fluorophore. **d** Fluorescent emission signal from the membranes incubated with Cy-5 fluorophore per peptide.

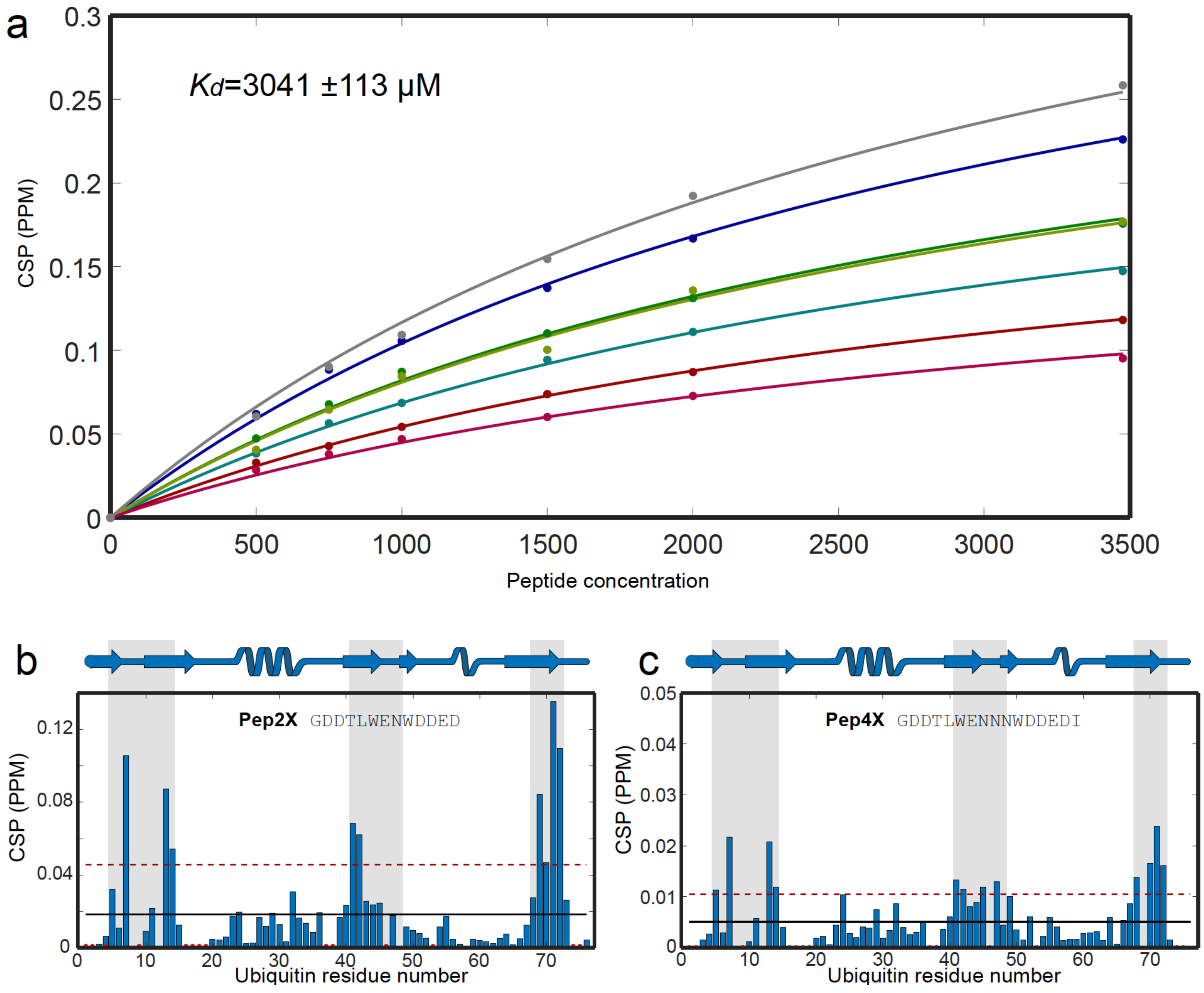

Fig. S2. Aromatic distance dependence in ubiquitin binding. **a** Quantification of binding of the Pep2X peptide to ubiquitin also shown in **b** as CSPs per residue. **c** CSP per residue for Pep4X. The concentration of the ubiquitin was 60 μM and the concentration of peptides 3.5 mM (Pep2X) and 3.2 mM (Pep4x).

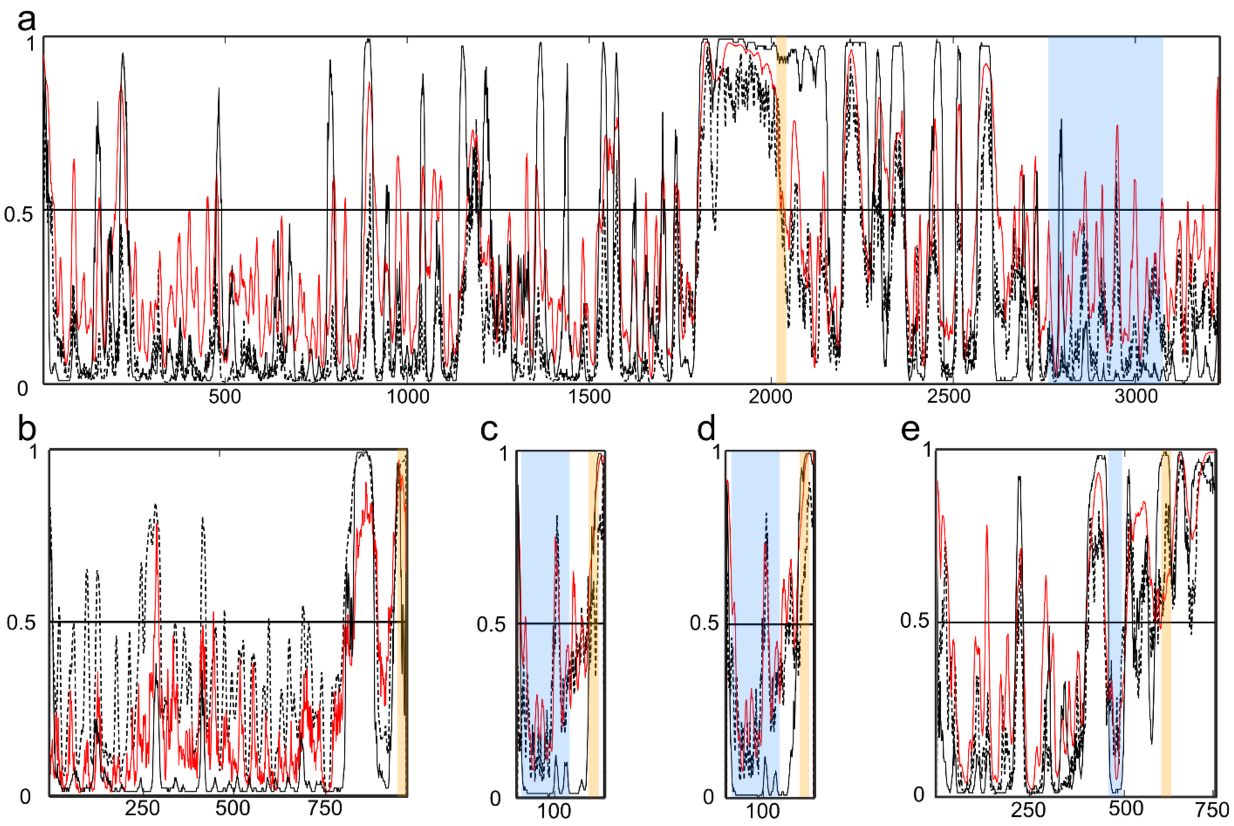

Fig. S3. Disorder predictions of proteins containing the DisUBM. The predicted disorder propensity of proteins containing DisUBMs was predicted using IUpred2 (https://iupred2a.elte.hu) (striped black), Pondr-fit VSL2 (http://www.pondr.com) (red) and Disopred3 with default settings (http://bioinf.cs.ucl.ac.uk/psipred) (black). The predicted disorder is plotted as a function of residue number. the DisUBM motif is indicated by the orange bars, and the nearest domain involved in ubiquitin signaling is indicated by the blue bars. **a** spPtr1, **b** spRPN2, **c** hUB2R1, **d** hUB2R2 and **e** hASCC2.

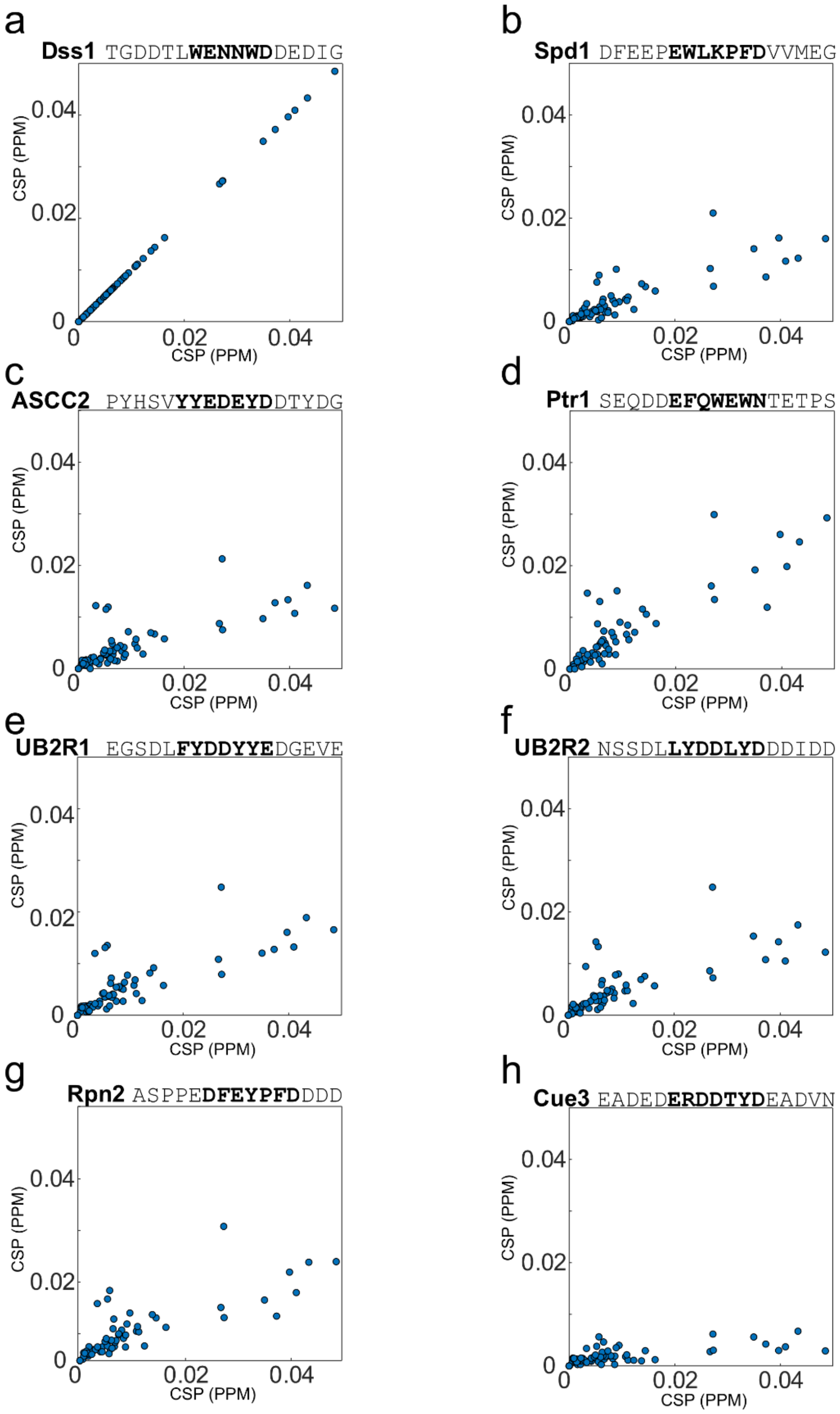

Fig. S4 CSP Correlations of DisUBM peptides

Scatter plots showing the correlation between the CSP of signals from ^{1}H-^{15}N-HSQC spectra of 100 µM ^{15}N-labeled ubiquitin with 1 mM Dss1 peptide on the x-axis and CSP signals of ubiquitin with 1 mM of the indicated peptide on the y-axis.

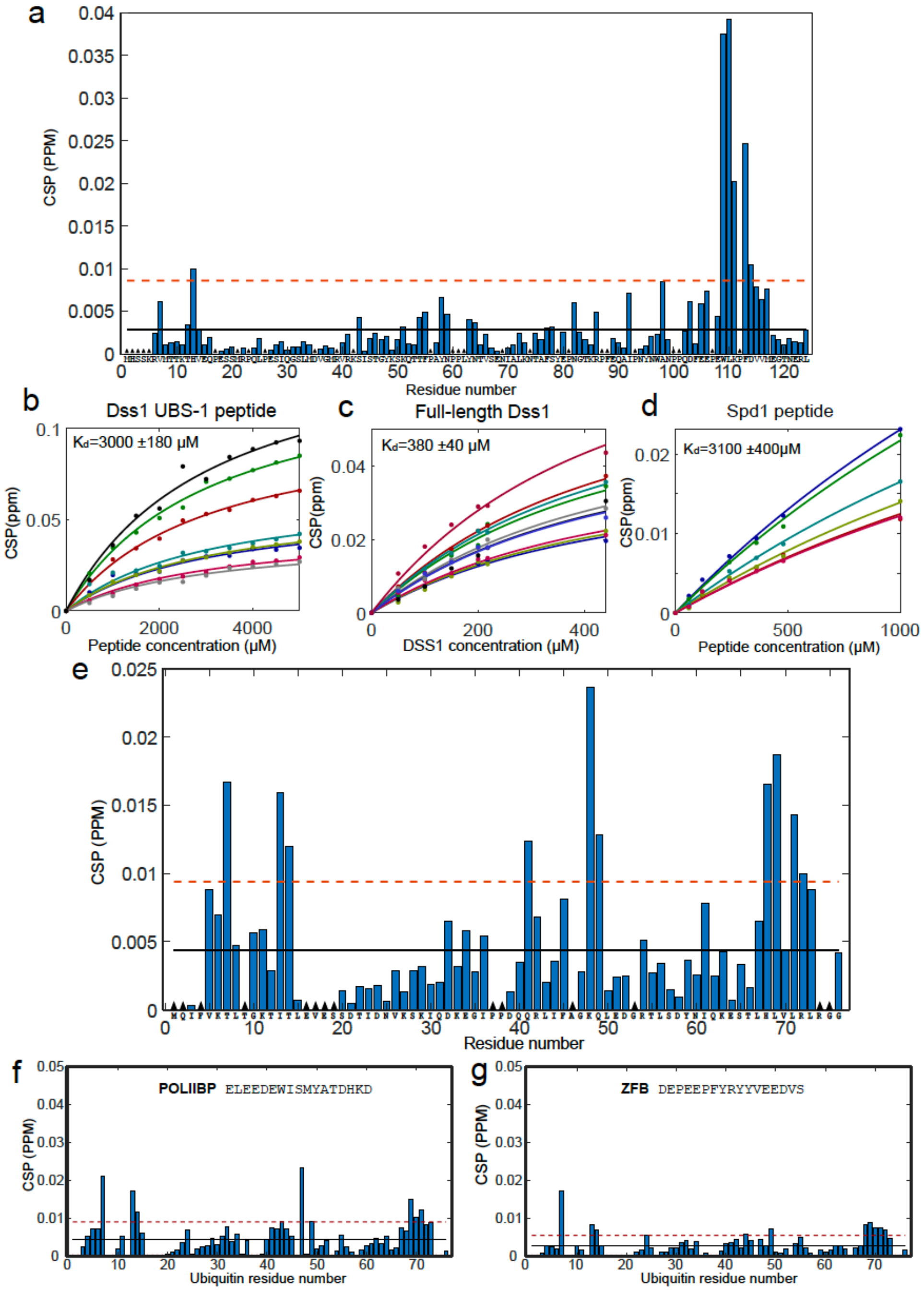

Fig. S5 NMR data from the interaction between ubiquitin, Dss1 and Spd1, and peptides from proteins ative in transcription. **a** CSP per residue of 30 μM ^{15}N-labeled Spd1 upon addition of 560 μM ubiquitin analyzed by ^{1}H-^{15}N-HSQC NMR. The black line is the average CSP, and the red dotted line is the average CSP + one standard deviation. **b-d** Changes in CSP by concentration of peptide or protein. Each fit line and corresponding colored data points reflect the CSPs of individual NMR signals from ^{1}H-^{15}N-HSQC spectra of 100 μM ^{15}N-labeled ubiquitin upon titration with either peptide or protein. All data points in one titration were fitted using a global fit to obtain the K_d values reported on each plot. **e** CSP per residue of 100 μM ^{15}N-labeled Ubiquitin upon addition of 640 μM Spd1-CTD analyzed by ^{1}H-^{15}N-HSQC NMR. The black line is the average CSP, and the red dotted line is the average CSP + one standard deviation. **f** CSP per residue of 120

μM ^{15}N-labeled Ubiquitin upon addition 1 mM POLIIBP peptide and **g** CSP per residue of 120 μM ^{15}N-labeled ubiquitin upon addition 1 mM ZBF peptide

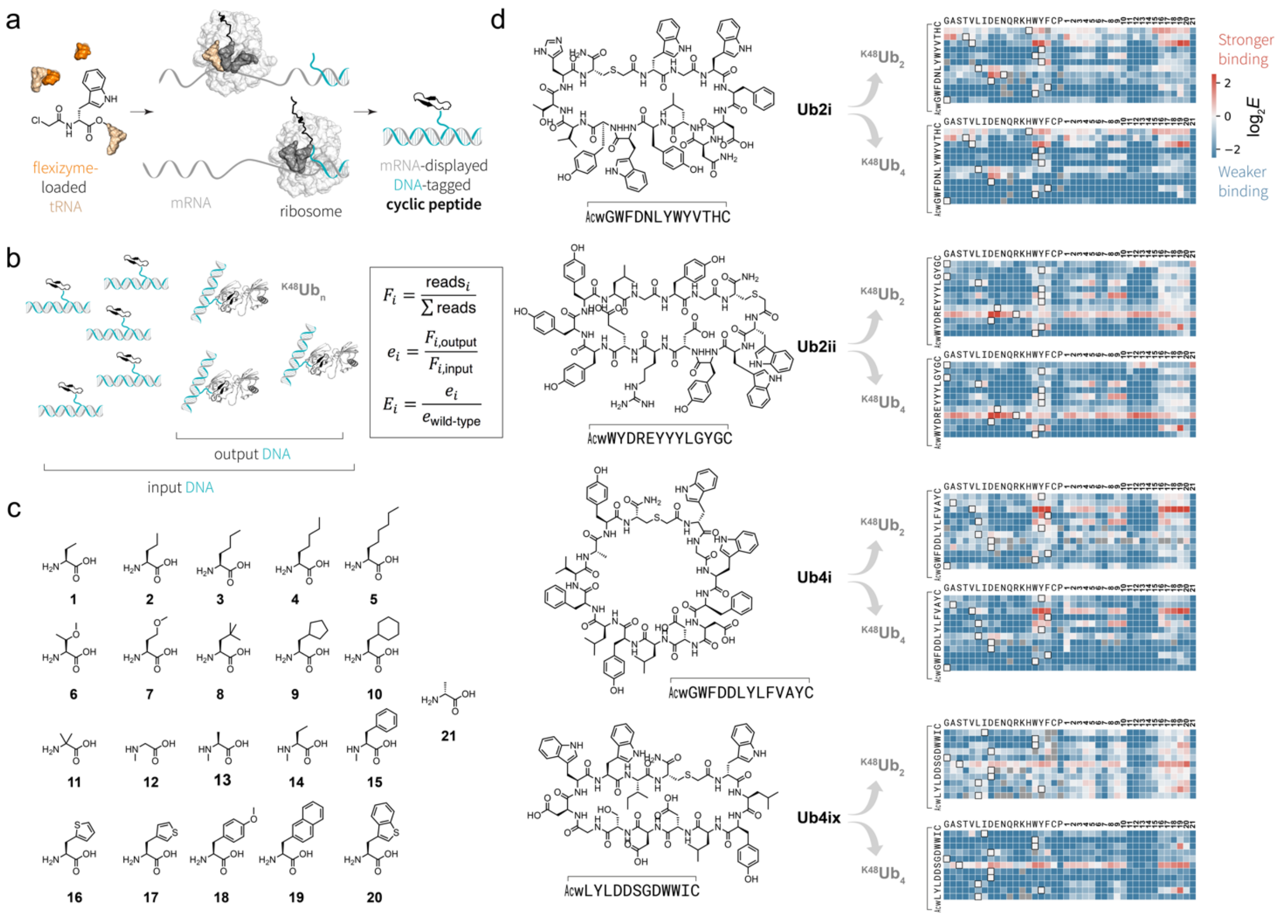

Fig. S6. Deep mutational scanning of ubiquitin chain binding *de novo* cyclic peptides.

a Genetic code reprogramming using flexizyme permits ribosomal peptide synthesis of cyclic peptides; initiating peptide synthesis with an N-modified chloroacetyl amino acid allows for spontaneous thioether bond formation with a cysteine in the same peptide chain. Coupled to mRNA display, each cyclic peptide can be tagged with its encoding DNA. **b** Deep mutational scanning of cyclic peptides. Saturation mutagenesis libraries (all possible single mutations) of DNA-tagged cyclic peptides can be assembled and sorted for binding to immobilized protein targets (in this case K48-linked ubiquitin chains). Populations of mutant cyclic peptides before and after binding ($F_{i,input}$ and $F_{i,output}$, respectively) can be calculated by counting next generation sequencing reads. Enrichment ratios (e_i) can be calculated for each individual mutant, which reports on relative binding strength to the protein target, which can then be normalized to the enrichment ratio of the wild-type mutant peptide (E_i). **c** Deep mutational scanning of cyclic peptides can be coupled to additional genetic code reprogramming to allow the analysis of mutations to non-canonical amino acids, such as the 21 shown. **d** Deep mutational scanning of cyclic peptides binding to K48-linked ubiquitin chains highlights essential amino acids and beneficial substitutions. Saturation mutagenesis libraries of cyclic peptides **Ub2i**, **Ub2ii**, **Ub4i**, **Ub4ix**, including non-canonical amino acids, were pooled and analyzed in parallel using the deep mutational scanning protocol, with either $^{K48}Ub_2$ or $^{K48}Ub_4$ as the target. Positive enrichment (E), red, corresponds to stronger binding than the wild type; Negative enrichment, blue, corresponds to weaker. Grey indicates that the peptide was not present in the output library.

Supplemental Note

Supplemental discussion of deep mutational scanning of ubiquitin chain binding *de novo* cyclic peptides. The relevance of this mutational scanning for the DisUBM is discussed in the main text. However, this data set contains other results worthy of discussion. In these experiments four ubiquitin chain-binding cyclic peptides **Ub2i**, **Ub2ii**, **Ub4i**, **Ub4ix** were subjected to site-saturation mutagenesis, with mutations to 19 canonical and 21 non-canonical amino acids (Supplemental **Fig. S5b**), and assessed for binding to two K48-linked ubiquitin chains $^{K48}Ub_2$, $^{K48}Ub_4$. Positive enrichment ratios (red) indicate stronger binding than the wild-type (unmutated cyclic peptide), whereas negative enrichment ratios (blue) indicate weaker binding (Supplemental **Fig. S5c**).

It is worth noting that, while most mutations weaken binding, beneficial mutations exist. Moreover, at certain sequences positions almost all mutations are beneficial (e.g. Arg in **Ub2ii**). Clearly the hit cyclic peptide sequences can contain non-optimal elements, despite the enormous size of the screened peptide library (~10^{12} unique sequences). This non-optimality is likely because the set of all possible sequences is even larger (~10^{15} sequences for 12 random amino acids) than the very large screening library.

Interestingly, mutations to cysteine are almost always deleterious, possibly because they induce the formation of cyclic peptides with incorrect ring size. Similarly, mutations to the non-canonical D-alanine (**21**) are almost always deleterious, likely because the cyclic peptide binding cannot tolerate changes to the backbone stereochemistry. An exception is the beneficial mutation of the C-terminal glycine of **Ub2ii** to D-alanine. Interestingly, such mutations have been tolerated in a different cyclic peptide protein system (CP2 binding to KDM4A[1,2]).

The patterns of enrichments are strikingly similar for **Ub2i**, **Ub2ii** and **Ub4i** binding to either $^{K48}Ub_2$ or $^{K48}Ub_4$, suggesting a similar binding mode to these two ubiquitin chain lengths. This confirms previous observations that **Ub2i**, **Ub2ii**, **Ub4i** bind $^{K48}Ub_2$ with a 1:1 stoichiometry and $^{K48}Ub_4$ with a 2:1 stoichiometry [3] i.e. that these cyclic peptides might bind the two di-ubiquitin units in the highly symmetrical structure of $^{K48}Ub_4$[4]. **Ub4ix** shows a sparser enrichment data set for $^{K48}Ub_2$, due to lower recovery of sequences in the output (after binding) sample. This is also consistent with previous observation that **Ub4ix** binds tightly to $^{K48}Ub_4$ with a 1:1 stoichiometry, and shows significantly weaker binding to $^{K48}Ub_2$[3].

Supplemental References

1 J. M. Rogers *et al.* Nonproteinogenic deep mutational scanning of linear and cyclic peptides. *Proc Natl Acad Sci U S A* **115**, 10959-10964, (2018)

2 A. Kawamura *et al.* Highly selective inhibition of histone demethylases by de novo macrocyclic peptides. *Nat Commun* **8**, 14773, (2017)

3 M. Nawatha *et al.* De novo macrocyclic peptides that specifically modulate Lys48-linked ubiquitin chains. *Nat Chem* **11**, 644-652, (2019)

4 M. J. Eddins *et al.* Crystal structure and solution NMR studies of Lys48-linked tetraubiquitin at neutral pH. *J Mol Biol* **367**, 204-211, (2007)

Supplemental Table S1. Agadir helix propensities of peptides in the PepSpot array

peptide number	Sequence	Predicted α-helix content (%)
1	GDDTLWENNWDDEDI	0,32
2	GDDTIWENNWDDEDI	0,26
3	GDDTVWENNWDDEDI	0,23
4	GDDTQWENNWDDEDI	0,59
5	GDDTFWENNWDDEDI	0,32
6	GDDTGWENNWDDEDI	0,24
7	GDDTAWENNWDDEDI	0,51
8	GDDTDWENNWDDEDI	0,32
9	GDDTEWENNWDDEDI	0,9
10	GDDTPWENNWDDEDI	0,25
11	GDDTWWENNWDDEDI	0,42
12	GDDTLDENNWDDEDI	0,26
13	GDDTLYENNWDDEDI	0,34
14	GDDTLFENNWDDEDI	0,3
15	GDDTLPENNWDDEDI	0,13
16	GDDTLAENNWDDEDI	0,56
17	GDDTLGENNWDDEDI	0,23
18	GDDTLLENNWDDEDI	0,47
19	GKKTLWENNWKKKKI	3,66
20	GDDTLWEDDWDDEDI	0,26
21	GNNTLWENNWNNENI	0,79
22	GDDTLWQNNWDDQDI	0,55
23	GEETLWENNWEEEEI	0,56
24	GDDTLWDNNWDDDDI	0,22
25	GDDDLWENNWDDEDI	0,29
26	GGGTLWGNNWGGGGI	0,08
27	GDDTLWKKKWDDEDI	7,15
28	GDDTLWENNYDDEDI	0,34
29	GDDTLWENNFDDEDI	0,32
30	GDDTLWENNPDDEDI	0,27
31	GDDTLWENNADDEDI	0,3
32	GDDTLWENNGDDEDI	0,22
33	GDDTLWENNLDDEDI	0,29
34	GDDTLWENNDDDEDI	0,22
35	GDDTLWENNEDDEDI	0,22
36	GDDTLWAAAWDDEDI	2,33
37	GDDTLWPNPWDDEDI	0,2
38	GDDTLWEVNWDDEDI	0,39
39	GDDTLWETNWDDEDI	0,27
40	GDDTLWEPNWDDEDI	0,18
41	GDDTLWENNWWDDED	0,27
42	DDTLWWENNWWDDED	0,41

43	DLWENNWDLWENNWD	0,43
44	EGSDLFYDDYYEDGE	0,19
45	EDPDLKAAIQESLRE	14,7
46	IQEDWELAERLQREE	29,69
47	LKVDVIDLTIESSSD	0,2
48	GEAEERVVVISSSED	0,77
49	MSRAALPSLENLEDD	0,33

Supplemental Table S2. List of proteins with potential DisUBM (see separate excel file)
